# Genotype-driven sensitivity of mice to tick-borne encephalitis virus correlates with differential host responses in peripheral macrophages and brain

**DOI:** 10.1186/s12974-025-03354-1

**Published:** 2025-01-28

**Authors:** Michaela Berankova, Jiri Holoubek, Vaclav Hönig, Zuzana Matusova, Martin Palus, Jiri Salat, Imtissal Krayem, Jarmila Vojtiskova, Pavel Svoboda, Veronika Pranclova, Lukas Valihrach, Peter Demant, Marie Lipoldova, Daniel Ruzek

**Affiliations:** 1 Department of Experimental Biology, Faculty of Science, Masaryk University, Brno, Czech Republic; 2 Laboratory of Arbovirology, Institute of Parasitology, Biology Centre of the Czech Academy of Sciences, Ceske Budejovice, Czech Republic; 3 Laboratory of Emerging Viral Diseases, Veterinary Research Institute, Brno, Czech Republic; 4 Laboratory of Gene Expression, Institute of Biotechnology of the Czech Academy of Sciences, Vestec, Czech Republic; 5 Faculty of Science, Charles University, Prague, Czech Republic; 6 Laboratory of Molecular and Cellular Immunology, Institute of Molecular Genetics, Czech Academy of Sciences, Prague, Czech Republic; 7 Faculty of Science, University of South Bohemia, Ceske Budejovice, Czech Republic; 8 Department of Cellular Neurophysiology, Institute of Experimental Medicine of the Czech Academy of Sciences, Prague, Czech Republic; 9 Department of Molecular and Cellular Biology, Roswell Park Comprehensive Cancer Center, Buffalo, NY USA; 10 Department of Medical Genetics, Faculty of Medicine, Charles University, 3rd Prague, Czech Republic; 11 Present Address: Institute of Organic Chemistry and Biochemistry, Czech Academy of Sciences, Prague, Czechia

**Keywords:** Tick-borne encephalitis, Tick-borne encephalitis virus, Mouse model, Neuroinflammation, Genetics, Transcriptomics, Macrophages

## Abstract

**Background:**

Tick-borne encephalitis (TBE) is the most common tick-borne viral infection in Eurasia. Outcomes range from asymptomatic infection to fatal encephalitis, with host genetics likely playing a role. BALB/c mice have intermediate susceptibility to TBE virus (TBEV) and STS mice are highly resistant, whereas the recombinant congenic strain CcS-11, which carries 12.5% of the STS genome on the BALB/c background, is more susceptible than BALB/c mice. In the present study, we employed these genetically distinct mouse models to investigate the host response to TBEV infection in both peripheral macrophages, one of the initial target cell populations, and the brain, the terminal target organ of the virus.

**Methods:**

TBEV growth and the production of key cytokines and chemokines were measured and compared in macrophages derived from BALB/c, CcS-11, and STS mice. In addition, brains from these TBEV-infected mouse strains underwent in-depth transcriptomic analysis.

**Results:**

Virus production in BALB/c and CcS-11 macrophages exhibited similar kinetics 24 and 48 h post-infection (hpi), but CcS-11 macrophages yielded significantly higher titers 72 hpi. Macrophages from both sensitive strains demonstrated elevated chemokine and proinflammatory cytokine production upon infection, whereas the resistant strain, STS, showed no cytokine/chemokine activation. Transcriptomic analysis of brain tissue demonstrated that the genetic background of the mouse strains dictated their transcriptional response to infection. The resistant strain exhibited a more robust cell-mediated immune response, whereas both sensitive strains showed a less effective cell-mediated response but increased cytokine signaling and signs of demyelination, with loss of oligodendrocytes.

**Conclusions:**

Our findings suggest that variations in susceptibility linked to host genetic background correspond with distinct host responses, both in the periphery upon virus entry into the organism and in the brain, the target organ of the virus. These results provide insights into the influence of host genetics on the clinical trajectory of TBE.

**Supplementary Information:**

The online version contains supplementary material available at 10.1186/s12974-025-03354-1.

## Background

Tick-borne encephalitis (TBE) is a significant viral infection transmitted primarily by ticks [1, 2]. The causative agent, tick-borne encephalitis virus (TBEV), belongs to the *Flaviviridae* family, genus *Orthoflavivirus* [3], and is endemic to large areas of Eurasia. TBE poses a considerable public health threat, with a clinical presentation ranging from asymptomatic or mild flu-like symptoms to severe neurological complications and, in some cases, death [4, 5]. The clinical course of TBE typically progresses through three phases: an initial febrile phase, a period of remission, and neurological involvement characterized by symptoms such as meningitis, encephalitis, or meningoencephalitis [5]. Despite the availability of vaccines and advances in medical care, TBE continues to cause significant morbidity and mortality in affected regions. Understanding the pathogenesis of TBE, including host-virus interactions and immune responses, is crucial to developing effective prevention strategies and therapeutic interventions.

**Fig. 1:**
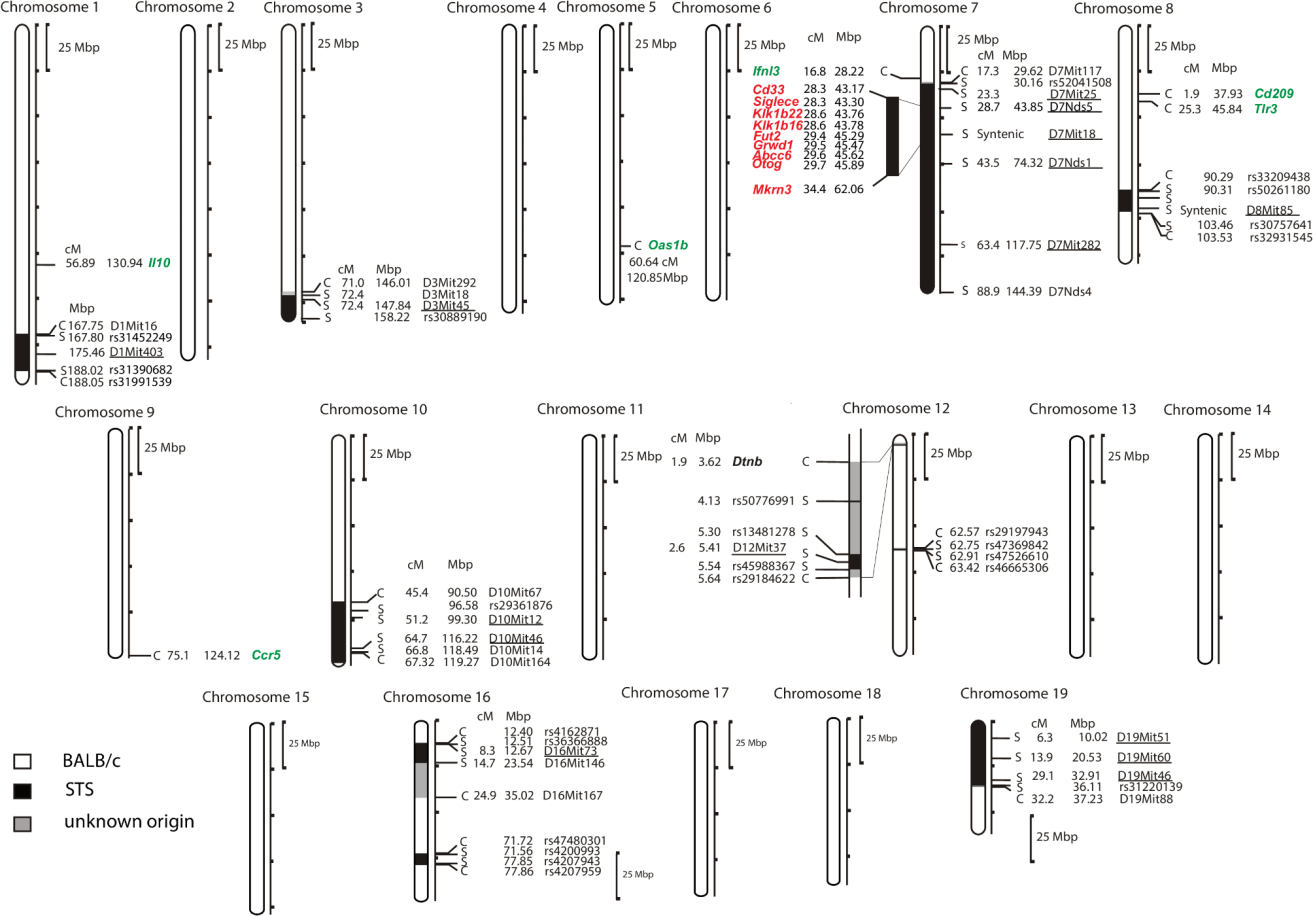
Updated version of the genetic map from Palus et al. [8] of the CcS-11 strain. STS regions are dark and white indicates BALB/c origin, whereas boundary regions of uncertain origin are shaded. The map includes only markers or SNPs that define the boundaries of the STS-derived segments and those tested for linkage (underlined). Genes known to influence TBEV susceptibility, such as *Oas1b*,* Cd209*,* Tlr3*,* Ccr5*,* Ifnl3*, and *Il10*, are highlighted in green. Potential candidate genes that may affect TBEV susceptibility (i.e., *Cd33*,* Klk1b22*,* Siglece*,* Klk1b16*,* Fut2*,* Grwd1*,* Abcc6*,* Otog*, and *Mkrn3)* are shown in red

To explore the impact of host genotype on susceptibility to TBEV infection and the resulting pathogenesis, we established a mouse model with three distinct strains: parental strains BALB/c and STS and the recombinant congenic strain CcS-11, which contains 12.5% of the STS genome on the BALB/c background [6, 7] (an updated version of the previous genetic map [8] is shown in Fig. 1). BALB/c mice demonstrate intermediate susceptibility to TBEV, whereas STS mice exhibit high resistance. Notably, CcS-11 mice have heightened susceptibility surpassing that of BALB/c mice. Upon subcutaneous inoculation, STS mice present with reduced and delayed viremia, decreased brain virus production, and muted cytokine/chemokine mRNA expression but mount a robust neutralizing antibody response. Conversely, the most susceptible strain, CcS-11, fails to produce neutralizing antibodies but exhibits pronounced cytokine/chemokine mRNA expression in the brain. Upon intracerebral inoculation, all strains are sensitive to infection, with active virus replication in the brain, yet STS mice exhibit significantly prolonged survival compared to CcS-11 mice. Furthermore, these strains exhibit differential expression of key cytokines/chemokines in the brain, notably interferon gamma-induced protein 10 (IP-10/CXCL10) and monocyte chemotactic protein-1 (MCP-1/CCL2) [6].

To pinpoint the location of the STS genes contributing to the susceptibility observed in CcS-11 mice, we previously assessed the survival outcomes of TBEV-infected F2 hybrids generated from crosses between the BALB/c and CcS-11 strains. Our investigations unveiled a novel locus on chromosome 7, linked to marker D7Nds5 (44.2 Mb), suggesting its involvement in controlling survival following TBEV infection. Further analysis of this locus to identify polymorphisms between the BALB/c and STS strains that affect RNA stability and gene function revealed nine potential candidate genes: *Cd33*, *Klk1b22*, *Siglece*, *Klk1b16*, *Fut2*, *Grwd1*, *Abcc6*, *Otog*, and *Mkrn3* (Fig. 1). Intriguingly, one of these genes, *Cd33*, harbored a nonsense mutation in the STS strain, suggesting a potential role in modulating susceptibility to TBEV [8].

Upon viral entry during tick feeding, the virus initially replicates in subcutaneous tissues [9]. Langerhans cells then transport the virus to lymph nodes, where it replicates in macrophages, initiating viremia [10]. As one of the first cell types to be infected, macrophages play a crucial role in the early stages of viral infection, potentially priming the organism for progression of the infection. Macrophages are pivotal to regulating an animal’s susceptibility to viral infections by monitoring key body compartments and controlling viral entry into critical target organs [11, 12]. Furthermore, macrophage polarization (M1/M2) significantly influences the host immune response [13].

The brain is the main target organ of TBEV, and infection leads to several pathological changes [14–16], including direct neuronal damage and death, immune cell infiltration into brain tissue, blood-brain barrier disruption, microglial activation, and demyelination [14–16]. We utilized large-scale gene expression arrays to identify and compare cellular genes and signaling pathways activated in the brains of mice with differing susceptibility to TBEV infection. Understanding these genes and pathways may elucidate the mechanisms underlying TBE pathogenesis within the CNS and highlight potential therapeutic targets for treating flavivirus-induced CNS diseases.

In the present study, we employed the previously developed animal model to investigate the differential pathogenesis of TBEV infection in both the periphery and brains of hosts with varying susceptibility to the infection. We explored whether macrophages derived from mice with different susceptibility to infection reflect the overall sensitivity of the organism. This study contributes to a deeper understanding of the molecular and cellular mechanisms driving TBEV pathogenesis. By elucidating the role of host genetic factors and the immune response in disease progression, we aim to identify potential biomarkers for susceptibility and novel targets for therapeutic intervention.

## Methods

### Viruses and cells

TBEV strain Hypr (European subtype; GenBank U39292.1) was passaged five times in the brains of suckling mice and once in BHK-21 cells before being utilized in this study. This strain was supplied by the Collection of Arboviruses, Biology Centre of the Czech Academy of Sciences (https://arboviruscollection.bcco.cz). Similarly, TBEV strain Neudoerfl (European subtype; GenBank U27495.1) was passaged four times in the brains of suckling mice, as well as in UKF-NB-4 and BHK-21 cells, before its use in this study. This strain was generously provided by Prof. Franz X. Heinz of the Medical University of Vienna.

Porcine kidney stable (PS) cells were cultured at 37 °C in Leibovitz (L-15) medium with L-glutamine, supplemented with 3% fetal bovine serum (FBS), 100 U/mL penicillin, and 100 µg/mL streptomycin (Sigma-Aldrich, Czech Republic). This cell line was provided by the National Reference Centre for Cell Cultures at the National Institute of Public Health, Prague, Czech Republic. Human lung adenocarcinoma A549 cells (ATCC CRM-CCL-185) were cultured in Dulbecco’s Modified Eagle Medium (DMEM) containing 10% FBS, 1% penicillin, 1% streptomycin, and 1% glutamine at 37 °C in a humidified atmosphere with 5% CO_2_.

### Mice

Specific pathogen-free adult female BALB/c, CcS-11, and STS mice, originally developed at the Netherlands Cancer Institute by Professor Peter Démant, were obtained from the breeding colony of the Institute of Molecular Genetics, Czech Academy of Sciences, Prague, Czech Republic, for use in the experiments. The mice were approximately 9 weeks old at the time of infection and housed in plastic cages with wood-chip bedding in a specific pathogen-free room maintained at a constant temperature of 22 °C and relative humidity of 65%. They were provided with sterilized pellet diet and water ad libitum.

### Macrophage isolation and infection

**Fig. 2:**
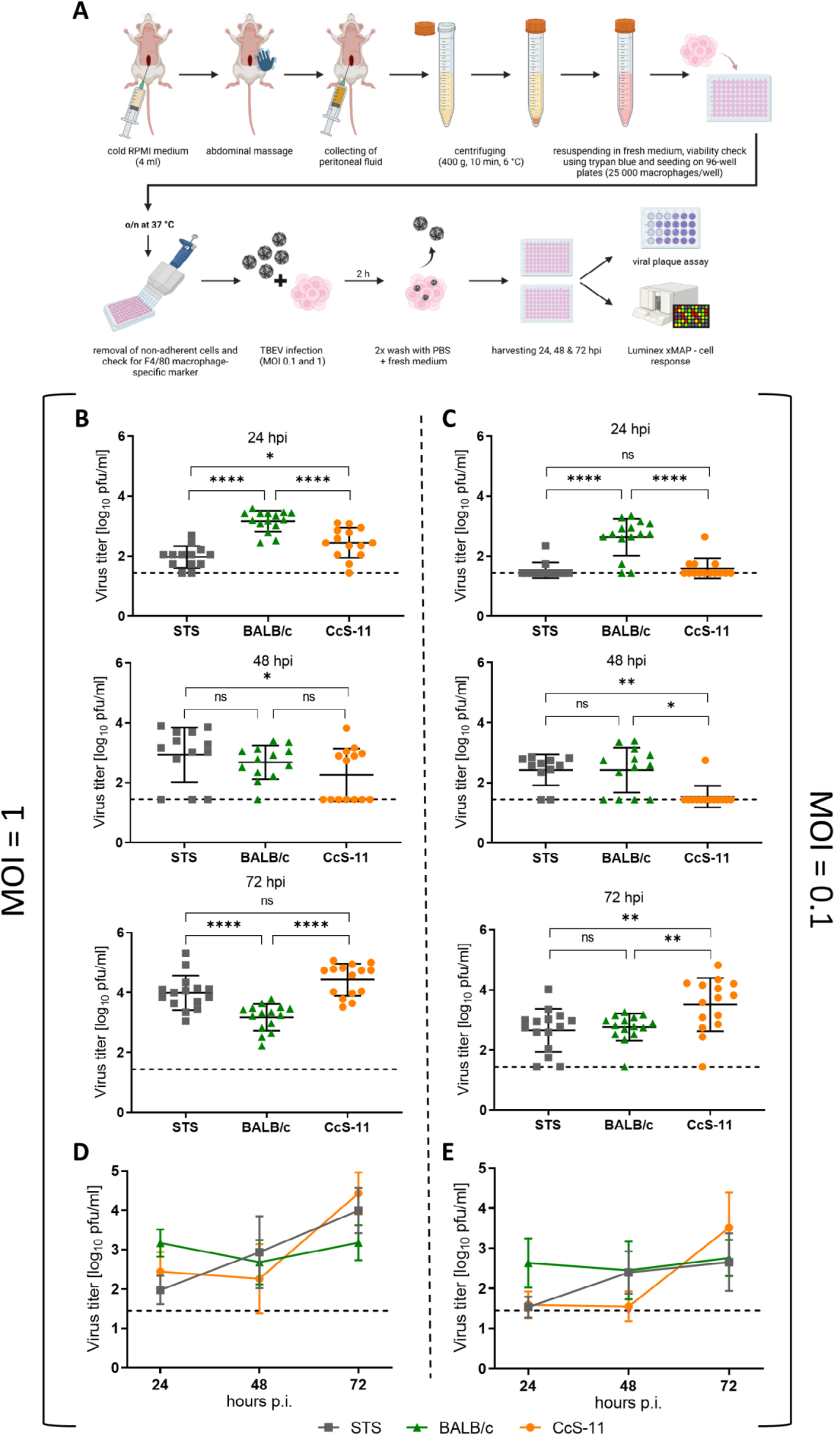
(**A**) Schematic representation of the experimental procedure used to isolate peripheral macrophages from BALB/c, STS, and CcS-11 mice, which were subsequently infected with TBEV. Supernatants were collected from both infected and control macrophages and analyzed by plaque assays and cytokine/chemokine measurements. (**B-C**) TBEV infection was achieved using the TBEV Hypr strain with a multiplicity of infection (MOI) of 1 (**B**) or 0.1 (**C**). Cell culture media was collected 24, 48, and 72 h post-infection (hpi) and subjected to plaque assays. (**D-E**) Dynamics of TBEV production from macrophages derived from BALB/c, STS, and CcS-11 mice infected with TBEV at MOI 1 (**D**) or 0.1 (**E**). ns: non-significant; *P < 0.05; **P < 0.01, ****P < 0.0001

Peritoneal exudate cells were recovered from mice by lavage with cold RPMI 1640 (Sigma-Aldrich, Prague, Czech Republic), washed once by centrifugation at 400 × g for 10 min, and resuspended in RPMI 1640 medium supplemented with 5% FBS, 100 U/mL penicillin, 100 U/mL streptomycin, and 2 mM glutamine (Sigma-Aldrich, Prague, Czech Republic). Cells were seeded on 96-well plates (25,000 cells/well). After a 24-h incubation at 37 °C and 5% CO_2_, the non-adherent cells were washed out. The purity of the macrophages was verified using rabbit anti-F4/80 antibody (1:100, Thermo Fisher Scientific) and goat anti-rabbit antibody Alexa Fluor 647 (1:1000, Thermo Fisher Scientific). Nuclei were stained with DAPI. Fluorescence microscopy was performed using the ImageXpress Pico automated imaging system, with CellReporterXpress software (Molecular Devices, USA) for image acquisition and analysis. The purity of the F4/80-positive cells was approximately 95%. Thereafter, TBEV infection was achieved with the TBEV Hypr strain (MOI 0.1 and 1). After a 2-h incubation, the cultures were washed twice with PBS and fresh medium added. The media and cell samples were harvested 24, 48, and 72 h post-infection (hpi) and subjected to plaque assay and multiplex cytokine bead array assay (Fig. 2A).

### Plaque assay

PS or A549 cells were used to determine virus titers following a modified version of a previously described protocol [17]. Ten-fold dilutions of virus samples were added to 24-well tissue culture plates, and PS or A549 cells were added in suspension (1.2 × 10^5^ cells per well). After a 4-h incubation, the suspension was overlaid with carboxymethylcellulose (1.5% in L-15 or DMEM medium). The plates were incubated for 5 days at 37 °C (with 5% CO_2_ if A549 cells were used), washed with PBS, and stained with naphthol blue-black (Sigma Aldrich). Virus titers were expressed as plaque forming units (pfu) per milliliter.

### Multiplex cytokine bead array assay

Concentrations of 48 chemokines, cytokines, and other regulatory factors were determined in supernatants from 24 and 72-h TBEV-infected and uninfected control macrophage cell cultures using the ProcartaPlex™ Mouse Immune Monitoring Panel (Thermo Fisher Scientific) and MagPix device (Luminex) as described previously [18]. Briefly, culture supernatants were cleared by centrifugation and 25 µL used for the analysis without previous inactivation. The samples were processed according to the manufacturer’s instructions using the overnight incubation protocol. Concentrations were determined in five biological replicates per interval for STS-derived cultures and six biological replicates per interval for CcS-11 and BALB/c-derived cultures.

### Intracerebral mouse infection

Adult mice of all three strains were infected with 10 pfu of TBEV (strain Neudoerfl) via intracerebral inoculation. At 6 dpi, when the infected mice exhibited neurological symptoms, the mice were sacrificed. Brains were removed from both mock- and virus-infected mice, individually weighed, and prepared as 20% (w/v) suspensions in phosphate-buffered saline (PBS) using a TissueLyser II (Qiagen).

### RNA purification

To analyze inborn differences in the RNA expression of select genes in uninfected mice, RNA was prepared by lysing a quarter of an organ (brain, spleen, or liver) and stored at -80 °C in TRI reagent (Sigma Aldrich). For microarray analysis, brain homogenates were clarified by centrifugation at 14,000 × g for 10 min at 4 °C. RNA was isolated using the TRIzol Plus RNA Purification Kit (Thermo Fisher, 12183555) according to the manufacturer’s instructions.

### Real-time quantitative RT-PCR

For the experiments to analyze inborn differences in the RNA expression of select genes, 1 µg of RNA was treated with DNase (Promega, M6101) and reverse-transcribed using 100 units of M-MLV Reverse Transcriptase (Sigma, M1302) with 1x MLV reverse transcriptase buffer, 1.4 µM of random hexamers (Thermo Fisher, N8080127), 2.5 units of ribonuclease inhibitor (Thermo Fisher, 15518012), and 5 mM of each dNTP (Sigma, DNTP100) per sample to obtain cDNA. The samples were then amplified as described previously [19, 20]. The cDNA was diluted 5-fold and 3 µL used for amplification by PCR: 3 min denaturation at 95 °C, 15 s denaturation at 95 °C, and 60 s annealing/extension at 60 °C, repeated 45 times, followed by a melt curve from 55 °C to 95 °C in 0.5 °C increments. Primers for the genes of interest were designed by Quantprime [21] (Supplementary Table 1), and iQ SYBR Green Supermix (Bio-RAD, 1708882) was used for quantification. *GAPDH* served as the internal control. Reactions were performed in a 384-well plate using an LC480II light cycler (Roche Molecular Systems, Inc.).

### Microarray analysis

Affymetrix Mouse Gene 1.0 ST arrays (Santa Clara, CA) were used for microarray analysis of RNA extracted from virus-infected and mock-infected brains. For each mouse strain, RNA from three virus-infected mice and two or three mock-infected mice was loaded onto individual microarrays according to the manufacturer’s specifications. All microarray experiments and initial analyses were performed at the microarray core facility (Institute of Molecular Genetics, Academy of Sciences of the Czech Republic). Data were background-corrected using the RMA method, which includes quantile normalization and variance stabilization using base-2 logarithmic transformation. The probe level signal was summarized at the gene level using the annotation provided in the package mogene10sttranscriptcluster.db. The LIMMA package (v. 3.14.4 [22]), was then used for statistical analysis. For the principal component analysis (PCA), unmapped probes were removed from the normalized data (~ 14% of probes), the mean signal of probes mapping to the same gene was calculated, and only the top 500 variable genes were used in the PCA (Supplementary Table 2A). The results of the differential expression analysis (DEA) were also filtered; unmapped probes and probes mapping to one gene but with inconsistent regulation were removed. Probes with the lowest false discovery rate (FDR) were selected as representative of probes mapping to the same gene. The significance threshold for differentially expressed genes (DEGs) was set to |log2FC| > 1 and FDR < 0.05. R package clusterProfiler (v. 4.4.4 [23]), and function *enrichGO (OrgDb = org.Mm.eg.db*,* ont = “ALL”*,* pAdjustMethod = “fdr”*,* pvalueCutoff = 0.1*,* minGSSize = 3)* were used for Gene Ontology [24, 25] overrepresentation analysis, with the adjusted *P*-value threshold set to 0.1 and all mapped genes as the parameter *universe*. The data were deposited in NCBI’s Gene Expression Omnibus [26] and are accessible through GEO Series Accession number GSE276086.

### Statistical analysis

The concentration data from the multiplex cytokine bead array assay did not follow a normal distribution despite several normalization attempts. Consequently, we used multiple Mann-Whitney non-parametric tests with two-stage step-up multiple comparison correction (Benjamini, Krieger, and Yekutieli; Q = 1%) to compare analyte concentrations between infected and control cultures, different intervals, and mouse strains. All other data, unless otherwise specified, were evaluated using the Mann–Whitney U test. Analyses were conducted using GraphPad Prism 7.04 (GraphPad Software, Inc., USA). *P*-values < 0.05 were considered significant.

## Results

### Growth kinetics of TBEV in macrophages derived from mice with varying susceptibility demonstrate distinct patterns

To investigate the susceptibility of mouse macrophages to TBEV infection, we isolated peritoneal macrophages from all three mouse strains (Fig. 2). Macrophage identity was confirmed through bright field microscopy and immunofluorescent staining against the macrophage-specific marker F4/80, which indicated that the majority of isolated cells were indeed macrophages (Supplementary Fig. 1).

Subsequently, the macrophages were infected with TBEV at MOI 1 and 0.1, and viral growth was determined by plaque assay 24, 48, and 72 hpi. At 24 hpi with MOI 1 (Fig. 2B), viral titers were significantly lower in STS and CcS-11 macrophages than in BALB/c macrophages (*P* < 0.0001). CcS-11 macrophages exhibited slightly higher titers (*P* < 0.05) than STS macrophages. However, at 48 hpi, a significant increase (*P* < 0.05) in the virus titer was observed in STS-derived macrophages compared to CcS-11-derived macrophages, whereas no changes were noted between BALB/c-derived macrophages and STS or CcS-11-derived macrophages. Notably, CcS-11 macrophages had the highest titers 72 hpi, indicating a high susceptibility to TBEV infection. Surprisingly, STS-derived macrophages also had similar titers despite STS mice being resistant to infection. Conversely, the viral titers of BALB/c-derived macrophages were lower than those of STS- and CcS-11-derived macrophages (*P* < 0.0001). The infection kinetics showed steady growth in STS-derived macrophages and a prolonged lag phase up to 48 hpi in BALB/c- and CcS-11-derived macrophages, followed by abrupt growth between 48 and 72 hpi (Fig. 2D).

The sensitivity of macrophages to a lower infectious dose (MOI 0.1) 24 and 48 hpi showed a comparable trend to that described above for MOI 1. However, at 72 hpi, virus titers of CcS-11-derived macrophages reached significantly higher values (*P* < 0.01) than the STS- and BALB/c-derived macrophages, whereas titers in the latter two groups were similar (Fig. 2C). A comparison of infection kinetics revealed a delayed increase in virus production, eventually reaching the highest titers in CcS-11-derived macrophages (Fig. 2E).

These findings demonstrate that virus production in macrophages from BALB/c and CcS-11 mice exhibit similar kinetics in the first 48 h after infection but results in dramatically higher titers in macrophages from highly susceptible CcS-11 mice 72 hpi. Conversely, macrophages from TBEV-resistant STS mice have a steady increase in virus titers over time.

### TBEV elicits differential immune responses in macrophages from mice differing in susceptibility to the infection

**Fig. 3:**
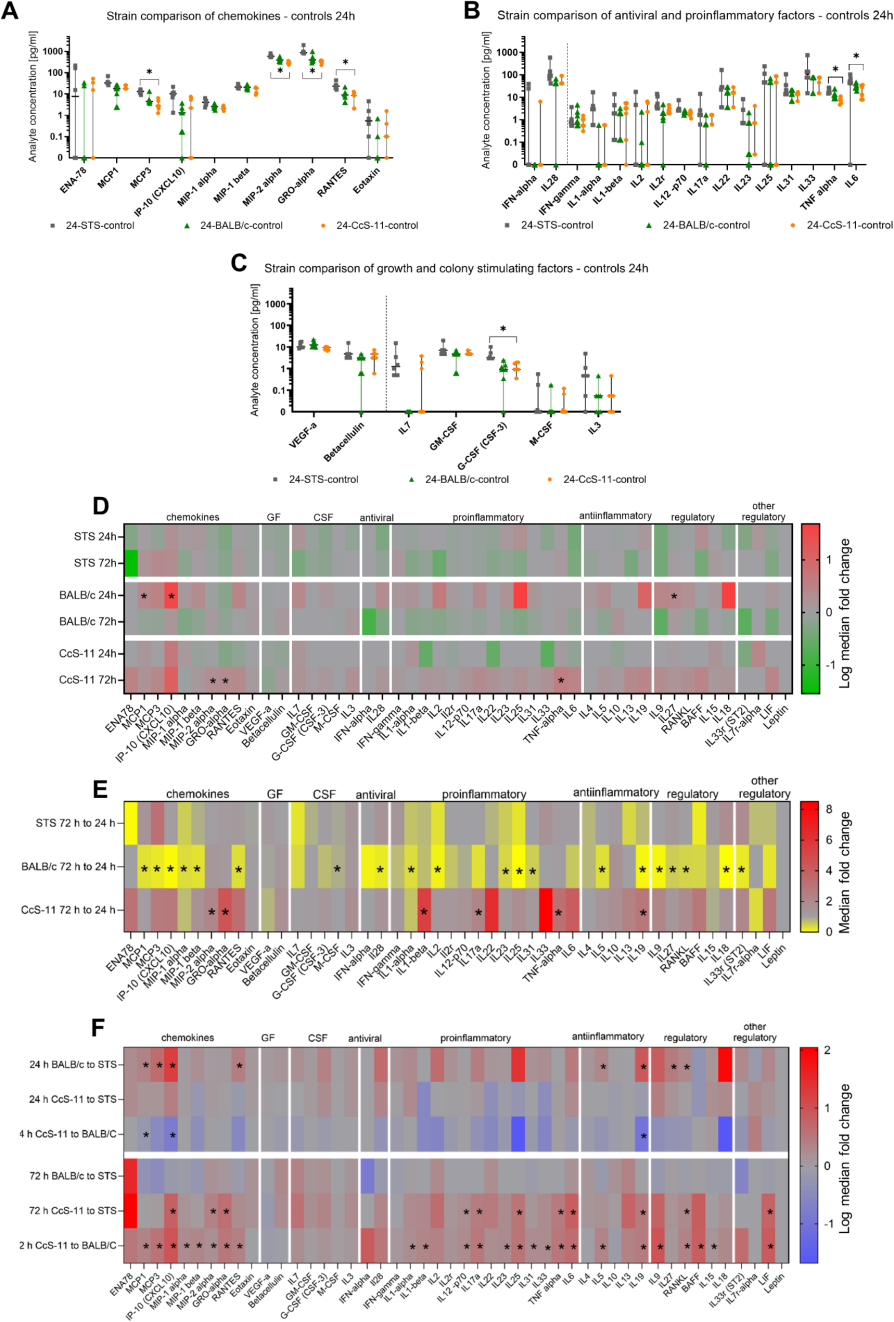
Analyte concentrations (**A-C**) and median fold changes in analyte concentrations (**D-F**) in supernatants of macrophage cultures. Concentrations were determined in five biological replicates for STS-derived cultures and six replicates for CcS-11 and BALB/c-derived cultures using Luminex MagPix technology. Significant differences based on the Mann-Whitney multiple comparison test with two-stage step-up multiple comparison correction (Benjamini, Krieger, Yekutieli; Q = 1%) are indicated by asterisks. Concentrations (median, 95% CI, logarithmic scale) of chemokines (**A**), antiviral and proinflammatory factors (**B**), and growth and colony-stimulating factors (**C**) in uninfected control cultures after 24 h of cultivation. (**D**) Log median fold changes in the concentration in supernatants from 24 and 72-h TBEV-infected STS, BALB/c, and CcS-11-derived macrophage cultures compared to uninfected controls. Red indicates an increase compared to the controls, and green indicates a decrease compared to the controls. (**E**) Median fold change in the concentration normalized to control in supernatant from 72-h TBEV-infected STS, BALB/c, and CcS-11-derived macrophage cultures compared to 24-h TBEV-infected cultures. Red indicates an increase at 72 h compared to 24 h post-infection, and yellow indicates a decrease compared to the 24-h interval. (**F**) Log median fold change in the analyte concentration normalized to control in supernatants from 24 and 72-h TBEV-infected macrophage cultures compared among the different mouse strains from which they were derived (STS, BALB/c, and CcS-11). Red indicates an increase in the concentration in the culture of the first strain compared to the second strain, and blue indicates a decrease. *P < 0.05

Activation of the immune response in macrophages derived from mice with different susceptibility to TBEV infection was analyzed in cell culture supernatants 24 and 48 hpi using multiplex cytokine bead array assays. Notably, we did not observe a significant change in the concentration of measured analytes in infected cultures derived from STS mice at either time interval compared to uninfected cells. In contrast, BALB/c-derived cultures exhibited a significant increase in IP-10 (CXCL10), MCP1, and IL-27 24 hpi, whereas upregulation of MIP-2 alpha, GRO-alpha, and TNF-alpha was observed 48 hpi in CcS-11-derived macrophage cultures compared to cultures of uninfected cells (Fig. 3D, Supplementary Figs. 2–6).

Interestingly, although uninfected STS-derived cultures showed no changes between the 24 and 72-h intervals (Supplementary Fig. 7), significant increases in multiple analytes were observed in both BALB/c and CcS-11-derived cultures. BALB/c cultures exhibited upregulation of several chemokines (IP-10, MSP1, MCP3, MIP-1 alpha, MIP-1 beta), antiviral and proinflammatory cytokines (e.g., IL-2, IL-17a), VEGF, and several regulatory factors (e.g., RANKL, IL-18, IL-33r) after 72 h of cultivation. Conversely, with the exception of VEGF, different analytes were upregulated in CcS-11-derived cultures 72 hpi, including MIP-2 alpha, GRO-alpha, RANTES, TNF-alpha, and IL-6 (Supplementary Figs. 8–9).

Regarding analyte dynamics in infected cultures, we did not observe significant changes for STS cultures. However, multiple analytes from all groups, except growth factors, were decreased in BALB/c cultures at 72 h compared to 24 h (Fig. 3D, Supplementary Figs. 10–12). In CcS-11 cultures, chemokines MIP2 alpha and GRO-alpha, as well as proinflammatory cytokines IL-1 beta, IL-17a, and TNF-alpha, were upregulated 72 hpi compared to the 24-h interval (Fig. 3D, Supplementary Fig. 9).

Significant differences were observed between uninfected macrophage cultures derived from STS and CcS-11 mice, particularly at 24 hpi. In particular, MCP3, MIP-2 alpha, GRO-alpha, G-CSF, RANTES, TNF-alpha, IL-6, and G-CSF were significantly decreased in CcS-11-derived macrophage cultures compared to STS-derived macrophage cultures (Fig. 3A-C).

The differences in analyte concentration between the individual strains were even more pronounced in infected cultures. At 24 hpi, BALB/c-derived cultures had significantly higher concentrations of chemokines MCP1, MCP3, IP-10, and RANTES; anti-inflammatory cytokines IL-5 and IL-19; and immune regulatory factors IL-27 and RANKL compared to STS-derived cultures. However, there were no significant differences between these strains 72 hpi. Conversely, there were no significant differences between CcS-11 and STS 24 hpi, whereas chemokines IP-10, MIP-2 alpha, and GRO-alpha; pro-inflammatory cytokines IL-12p70, IL-17a, IL-25, TNF-alpha, and IL-6; IL-19; RANKL; and LIF were upregulated in CcS-11 72 hpi. Furthermore, CcS-11 exhibited decreased concentrations of MCP1, IP-10, and IL-19 compared to BALB/c 24 hpi. However, almost all analyzed chemokines, pro-inflammatory and anti-inflammatory cytokines, and regulatory factors were upregulated in CcS-11-derived macrophages compared to BALB/c-derived cultures 72 hpi (Fig. 3F).

Therefore, STS-derived macrophages exhibited minor production of chemokines, cytokines, and other regulatory factors in response to TBEV infection, whereas BALB/c-derived cultures exhibited a faster reaction, which was particularly evident 24 hpi, with concentrations rapidly decreasing by 72 h. CcS-11-derived cells exhibited a delayed and less broad reaction to TBEV infection, becoming more pronounced at 72 h.

### Differential gene expression in organs of uninfected mice

Inborn differences in gene expression can influence susceptibility to infection. Therefore, we analyzed the mRNA expression of previously identified candidate genes *Cd33*,* Klk1b22*,* Siglece*,* Klk1b16*,* Fut2*,* Grwd1*,* Abcc6*,* Otog*, and *Mkrn3* in the brains, spleens, and livers of uninfected STS, BALB/c, and CcS-11 mice. We also examined the expression of tyrosine phosphatase genes *Shp1/Ptpn6* and *Shp2/Ptpn11*, as CD33 contains two conserved tyrosine motifs, including an ITIM crucial for the recruitment of SHP1 and SHP2 tyrosine phosphatases [27]. These phosphatases also play roles in other immune regulatory pathways [28].

**Fig. 4:**
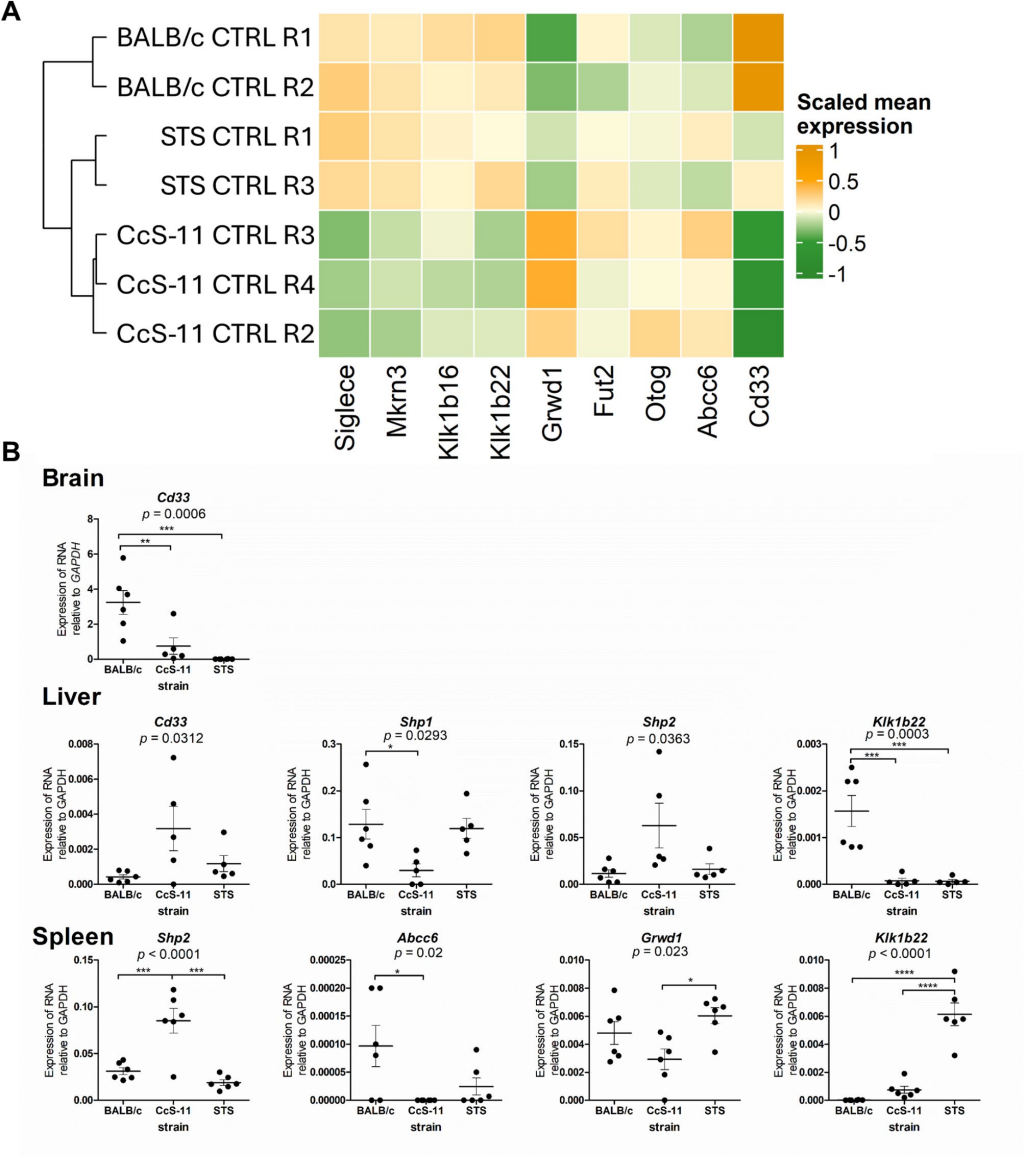
(**A**) Microarray-based transcriptomic analysis of the selected candidate genes influencing sensitivity to TBEV infection in the CNS of uninfected BALB/c, STS, and CcS-11 mice. (**B**) To examine inherent differences in the RNA expression of selected genes in uninfected BALB/c, CcS-11, and STS mice, RNA was extracted from the brain, spleen, and liver tissues and analyzed by RT-qPCR. Only genes and organs in which significant differences in expression were observed between the strains are presented. *P < 0.05; **P < 0.01, ***P < 0.001, ****P < 0.0001

Microarray-based transcriptomic analysis of the mouse brains revealed similar expression of the genes of interest in the brains of uninfected mice, with the exception of *Cd33* and *Grwd1*, and the expression did not change substantially after TBEV infection for any of the genes investigated (Fig. 4A). However, the expression of these candidate genes in uninfected mice was tissue-specific. RT-qPCR confirmed differential expression of *Cd33* in the brain (Fig. 4B), whereas differential expression of *Cd33* and *Klk1b22* was noted in the liver (Fig. 4B). In the spleen, *Abcc6*, *Grwd1*, and *Klk1b22* had differential expression (Fig. 4B).

Notably, the analysis of *Shp1/Ptpn6* and *Shp2/Ptpn11* expression yielded interesting results. *Shp1* expression was significantly lower in the livers of CcS-11 mice compared to BALB/c mice, whereas *Shp2* expression was higher than in both parent strains (Fig. 4B). Similarly, *Shp2* expression in the spleen was higher than in both parent strains (Fig. 4B). These findings are important because, in CcS-11 mice, *Shp1/Ptpn6* and *Shp2/Ptpn11* are located on BALB/c-derived segments of chromosomes 6 and 5, respectively.

Taken together, the differences in expression from BALB/c mice indicate gene interactions and perturbations of BALB/c pathways caused by the introduction of STS genes into the recombinant congenic strain CcS-11.

### Transcriptomic analysis revealed host genotype-dependent response to TBEV infection in brain

To identify transcriptional differences between the three tested mouse strains with and without TBEV infection, we performed gene expression profiling using Affymetrix 1.0 ST mouse whole-genome microarrays. To avoid peripheral responses to the infection, mice were inoculated with TBEV via the intracerebral route. At 6 dpi, when the infected mice exhibited neurological symptoms, they were sacrificed and their brains collected for viral titer quantification via plaque assay and for transcriptomic analysis. The plaque assay revealed high viral titers in all inoculated brains, ranging from 7 to 8.5 log_10_ pfu/g (Supplementary Fig. 15), with significantly higher titers in BALB/c mice compared to STS or CcS-11 mice (*P* < 0.001).

**Fig. 5:**
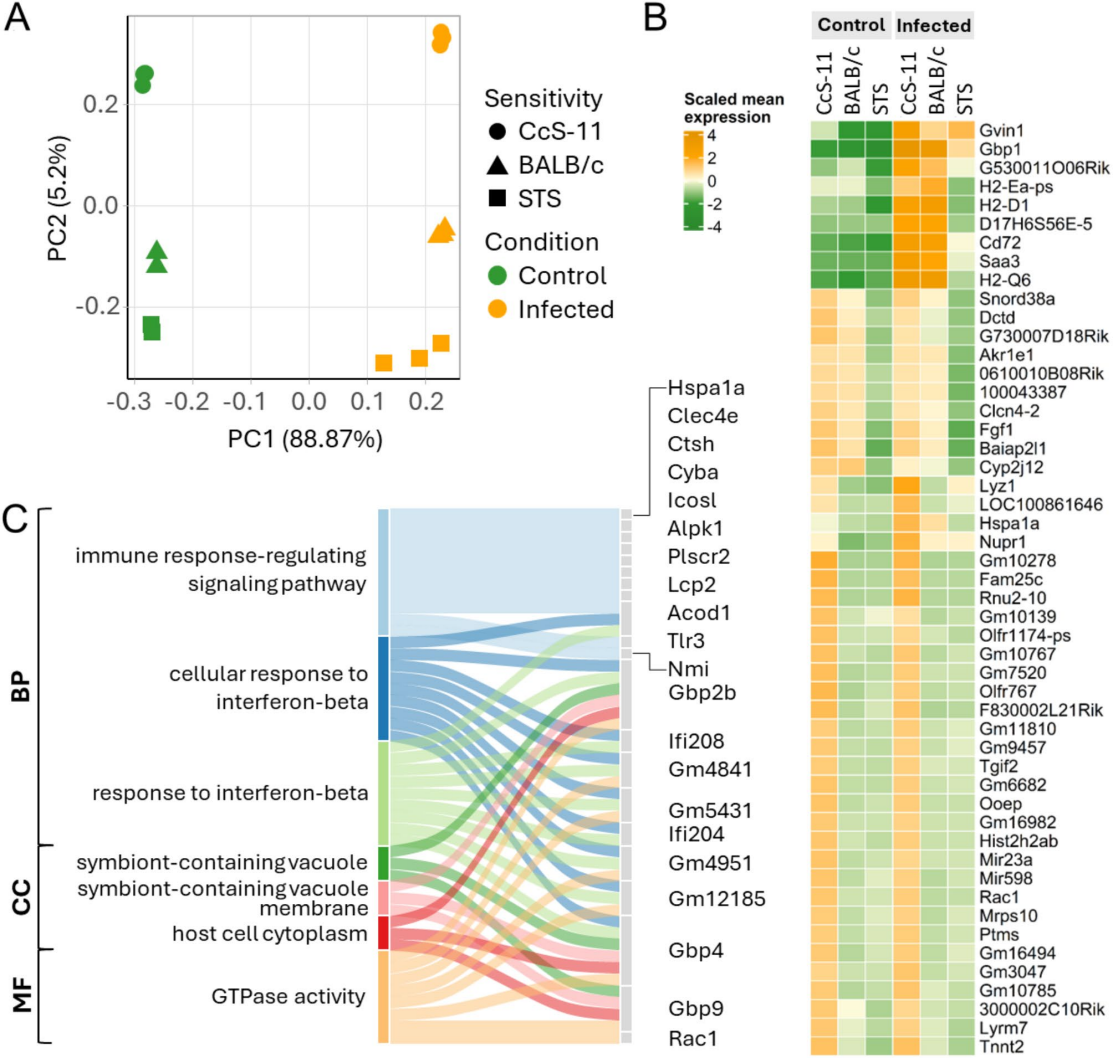
Microarray-based transcriptomic analysis of TBEV infection in the CNS of mice with different levels of susceptibility to the infection. (**A**) Principal component analysis revealed a separation of infected and control samples, as well as a transcriptional difference between mouse strains. More than 80% of the variability in the data is explained by the infection (PC1) and ~ 5% is explained by genotype (PC2). (**B**) The top 50 genes driving the genotype difference along the PC2 axis. (**C**) Overrepresentation analysis of the top 100 genes driving PC2, genotype-based separation (multiple testing correction by FDR, P_adj_ < 0.1). Genes enriched in each Gene Ontology term are listed. Abbreviations: BP – biological process, CC – cellular component, MF – molecular function

From the transcriptomic data, the initial PCA with the 500 most variable genes revealed a clear separation of infected and uninfected samples along the PC1 axis, explaining more than 80% of the variability in the dataset (Fig. 5A). The PC2 axis separated the samples based on their sensitivity to infection given their genetic background. Although the infection-resistant STS strain and moderately sensitive BALB/c strain clustered closer together, the highly sensitive CcS-11 strain was distant from them. Similar separation was also observed in a heatmap of the top 500 variable genes, with smaller groups of genes determining differences between mouse strains (Supplementary Fig. 16A). The genes were mostly related to various components of immune response cascades, such as the response to interferon-gamma, cytokine-mediated signaling pathways, or leukocyte-mediated immunity. Assessing the genes driving this difference along the PC2 axis not only in infected samples, but also in controls, we showed that immune response efficiency, namely the interferon-beta response and production represented by genes such as *Gbp2b*, *Gbp4*, *Ifi208*, *Ifi204*, and *Acod1*, distinguished the tested mouse strains at the transcriptional level (Fig. 5B-C, Supplementary Table 2B).

**Fig. 6:**
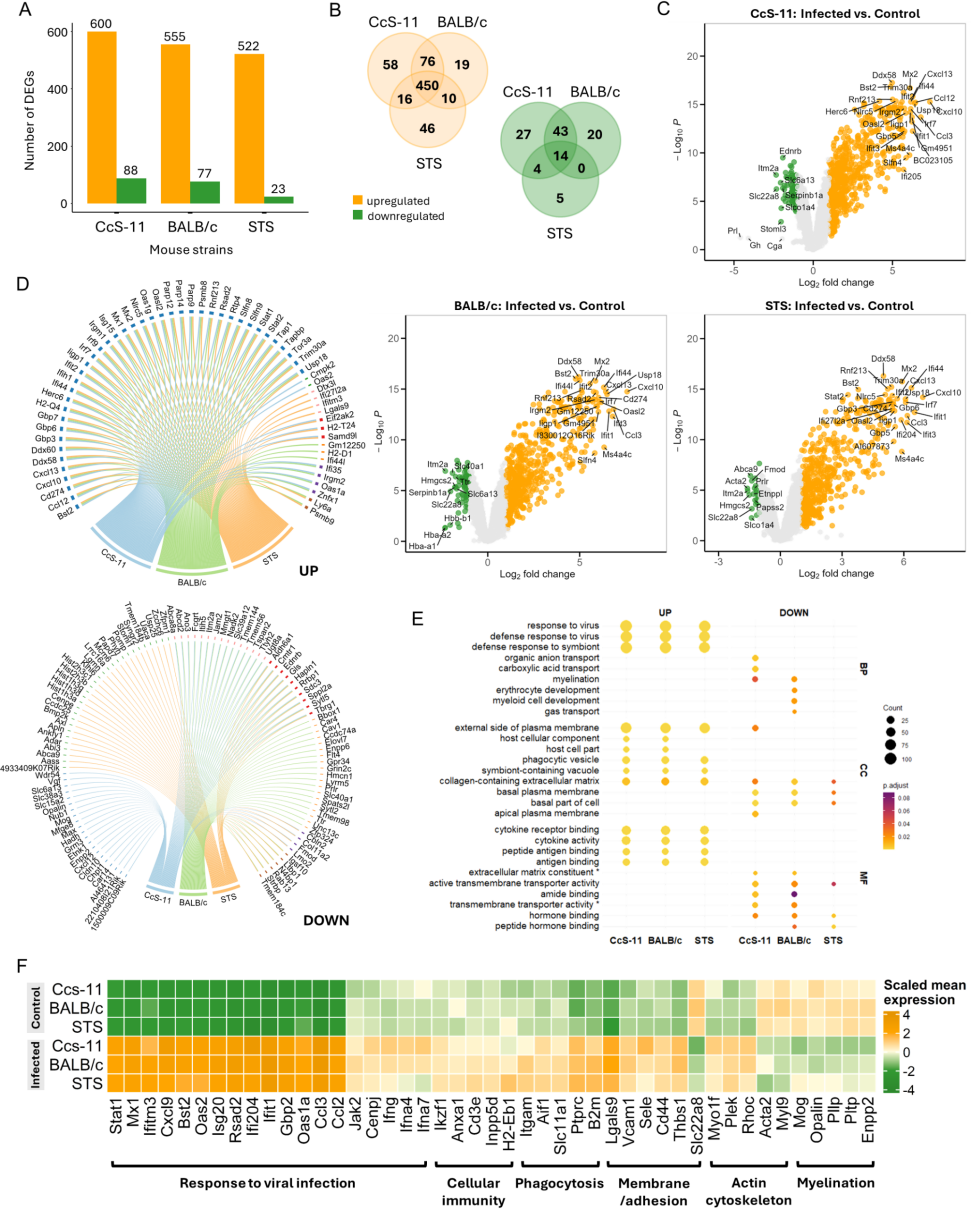
Transcriptomic analysis of TBEV infection in the CNS of mice with varying susceptibility. (**A**) Numbers of upregulated and downregulated differentially expressed genes (DEGs) in infected samples versus controls across all mouse strains analyzed. The significance thresholds were set to P_adj_ < 0.05 and |log_2_FC| > 1. (**B**) Venn diagrams of upregulated and downregulated unique and overlapping DEGs between the mouse strains. The 450 upregulated genes shared across strains represent the general immune response elicited upon infection in all strains. (**C**) Volcano plots showing DEGs in comparisons of infected and control samples across all mouse strains (|log2FC| >1, FDR < 0.05). (**D**) Top 50 DEGs of each strain between mock-infected control and TBEV-infected samples showing genes clustered in groups based on expression across strains. (**E**) Visualization of Gene Ontology overrepresentation analysis of DEGs, combining results across all mouse strains, showing unique and shared terms related to viral infection. The results were filtered for P_adj_ < 0.1, and results with count > 3 are displayed in the plot. (**F**) Heatmap of selected genes categorized based on their function and results from GO enrichment analysis of shared and unique genes. Mean and scaled expression values in replicates are shown. Abbreviations: BP – biological process, CC – cellular component, MF – molecular function

DEA revealed hundreds of DEGs in all tested mouse strains upon infection. Upregulated DEGs represented the majority of these changes in all strains, but we observed a decreasing trend in DEG numbers depending on the different sensitivities of the strains to infection (Fig. 6A-B, Supplementary Table 3A-C, 4A-C). Upregulation of the Stat1-mediated response to the virus, interferon response, immune system activation, and interferon-stimulated genes (ISGs; e.g., *Rsad2*,* IFITM3*,* Cxcl9*,* Bst2*, and *Oas2*) across all strains confirmed ongoing viral infection (Fig. 6C-F). The shared upregulated genes also included plasma membrane components and genes related to cell adhesion, actin cytoskeleton, phagocytosis, cytokine activity, and antigen binding (Fig. 6E-F). All mouse strains had significant downregulation of *Slc22a8* expression (Fig. 6F). Considering the role of chemokine signaling in the response to infection, we visualized individual components of this pathway using the KEGG database and their regulation in the three mouse strains upon infection. We found consistent regulation patterns in the brain across the mouse strains, suggesting that the primary differences between the mouse strains are due to their genetic backgrounds and the chemokine response following infection is similar across the mouse strains (Supplementary Fig. 16B).

We further focused on the genes characteristic of either the sensitive strains (CcS-11 and BALB/c) or the resistant strain (STS). Based on the Gene Ontology enrichment analysis, the upregulated genes unique to the resistant STS strain included genes involved in lymphocyte differentiation and activation (e.g., *H2-Eb1*, *Cd3e*). On the other hand, the sensitive strains upregulated cytokine signaling, specifically interferon alpha and gamma genes (*Ifna2*, *Ifna4*, *Ifna7*, *Ifna15*, *Ifng*) and regulators of JAK-STAT signaling (e.g., *Jak2*, *Cenpj*). Moreover, these strains downregulated oligodendrocyte genes involved in myelination (e.g., *Mog*, *Opalin*, *Pllp)*, suggesting that demyelination accompanies viral infection of the CNS in the sensitive strains (Fig. 6F).

These results demonstrate that genetic background shapes the transcriptional response to TBEV infection in mice. The resistant strain had a stronger cell-mediated immune response, whereas the sensitive strains had weaker cell-mediated immunity with demyelination, oligodendrocyte loss, and heightened cytokine signaling.

## Discussion

Studies in humans have increasingly shown that the host’s genetic background, along with other viral and host factors, plays a crucial role in determining TBEV susceptibility to infection and the clinical course of the disease [29–40]. Given that mice are valuable models for studying human TBE because they replicate the pathological and pathophysiological processes observed in severe human cases [41], we developed a mouse model that simulates different susceptibilities to TBEV based on host genotype [6, 8]. Previously, we demonstrated that the genotype-driven susceptibility to TBEV in these mice is associated with impaired production of neutralizing antibodies and an exaggerated cytokine/chemokine response in sensitive strains [6]. We also identified candidate genes that determine survival of these mice after TBEV infection [8].

Here, we demonstrated that the expression of these candidate genes varied among the strains in a tissue-specific manner. This suggests that certain tissues may have unique roles in mediating susceptibility or resistance to the virus. For example, higher expression of specific candidate genes in certain tissues could enhance the immune response, thereby providing protection, or contribute to a hyperinflammatory state that exacerbates disease severity. Conversely, lower expression in other tissues may lead to inadequate viral clearance and increased susceptibility. This suggests that the mice exhibit inherent differences even before infection, which could influence their initial response upon TBEV entry into the host organism. Notably, these mice also exhibit varying susceptibility to other pathogens and cancer, aligning with data obtained from other model systems [19, 20, 42–50]. However, it should be noted that the identified polymorphisms in the candidate genes affecting survival do not necessarily explain the differential host responses described in this study. It is likely that additional polymorphisms, yet to be identified, also contribute to these responses.

The crucial role of macrophages in determining an organism’s susceptibility to infection is well established. Macrophages are strategically positioned to monitor the body’s main compartments, enabling them to regulate the entry of viruses into target organs, thereby influencing the overall susceptibility to viral infections [11, 12]. Previously, we demonstrated that cultured mouse macrophages are susceptible to infection with the TBEV strain Hypr, a highly neuroinvasive and neurovirulent strain for laboratory mice, producing relatively high virus titers while remaining morphologically inactivated. In contrast, macrophages infected with the attenuated, non-neuroinvasive TBEV strain 263 did not demonstrate virus production but underwent clear morphological activation [51]. The inability of strain 263 to replicate in mouse macrophages, which serve as the first site of significant viral replication in vivo, may explain why this strain does not establish a serious infection in mice [52]. This suggests that macrophages play a crucial role in determining whether certain TBEV strains cause disease. However, whether macrophages derived from different host genotypes respond differently to the infection and if this variability explains why some genotypes are more susceptible to infection than others remains unknown. To address this question, we isolated peritoneal macrophages from mice with varying susceptibility to TBEV and characterized the viral growth and immune response in these cells. Virus production in macrophages from both susceptible mouse strains, BALB/c and CcS-11, followed similar kinetics, but we measured significantly higher titers in macrophages from the highly susceptible CcS-11 mice, despite a delay in virus production. In contrast, macrophages from TBEV-resistant STS mice exhibited a steady increase in virus titers over time. The higher virus titers in CcS-11 macrophages despite delayed virus production suggest that these cells may have an impaired ability to mount an effective antiviral response, potentially due to a genetic predisposition. In addition, STS-derived macrophages produce lower levels of chemokines, cytokines, and other regulatory factors in response to TBEV infection compared to both sensitive strains. These findings underscore the complexity of the host immune response to TBEV infection and highlight the pivotal role of macrophages in mediating this response. Previous proteome analysis of a mouse macrophage cell line infected with TBEV revealed that the expression of 265 host proteins was terminated during infection; these proteins were only detected in uninfected samples. Moreover, 73 newly synthesized proteins were identified in the infected cells [53]. However, protein expression patterns in the cultured macrophage cell line did not correlate with the patterns in macrophages cultured ex vivo [54]. Macrophages derived from STS mice produced higher levels of several soluble factors, including CCL5/RANTES, G-CSF, TNF alpha, and IL-6, than macrophages from CcS-11 mice even before infection. Following TBEV infection, STS macrophages did not show any significant activation of cytokine/chemokine expression, suggesting a level of preparedness that limits the infection without further immune response activation. In contrast, the low basal expression in CcS-11 macrophages was followed by an extensive immune response post-infection, but it still failed to efficiently limit virus growth. This all indicates a more controlled and perhaps more effective antiviral response in STS macrophages, which could be a key factor in the resistance of STS mice to TBEV. It remains unclear whether STS macrophages fail to respond to all stimuli or if their lack of responsiveness is specific to TBEV infection. Future experiments using exogenous cytokine-inducing agents could help determine whether these macrophages are generally capable of mounting a response and whether their unresponsiveness is uniquely associated with TBEV infection.

The brain is the main target of TBEV, with viral infection leading to diverse pathologies that dictate the clinical severity of the disease. In our mouse model, we previously showed that strains differing in susceptibility exhibit distinct patterns of mRNA expression of cytokines and chemokines. We also observed enhanced B-cell infiltration in the brains of resistant mice [6]. In this study, to further elucidate the brain’s response to infection, we conducted in-depth transcriptomic analysis of mouse brains from all three strains following TBEV infection. In contrast to our previous findings in which no differences in viral titers were observed in the brain between strains [6], we noted higher virus titers in the brains of BALB/c mice compared to CcS-11 or STS mice following intracerebral inoculation (Supplementary Fig. 15). This may be attributed to different mouse cohorts used in the previous and current study differing in age at the time of infection, or different virus stocks. The PCA distinctly separated the infected and uninfected samples and differentiated the samples based on their susceptibility to infection determined by their genetic background. Notably, the infection-resistant STS strain and moderately sensitive BALB/c strain clustered closely together, whereas the highly sensitive CcS-11 strain was noticeably distant from them. This observation confirmed our previous findings [6], highlighting unique gene expression profiles among the strains and their distinct responses to infection. Assessing the genes driving this difference not only in infected samples, but also in controls, revealed that all mouse strains significantly downregulated *Slc22a8*, the gene encoding the protein known as solute carrier family 22 (organic anion transporter), which is consistent with previous observations during flavivirus infection and reovirus CNS infection [55]. Solute carrier family 22, member 8 is crucial for the excretion and detoxification of endogenous and exogenous organic anions, particularly in the brain and kidney [56]. Upregulation of the Stat1-mediated response to the virus, interferon response, immune system activation, and ISGs, such as *Rsad2*,* IFITM3*,* Cxcl9*,* Bst2*, and *Oas2*, across all strains confirmed ongoing viral infection [57–60]. However, significant differences in strain-specific responses to the infection were also identified. In particular, immune response efficiency, namely the interferon-beta response and production represented by genes such as *Gbp2b*, *Gbp4*, *Ifi208*, *Ifi204*, and *Acod1*, distinguished the tested mouse strains at the transcriptional level. This was described previously in a genotypic analysis of these mouse strains and also at the protein level [6, 61]. These results collectively demonstrate that the genetic background of the mouse strains dictates their transcriptional response to infection. However, the specific genetic polymorphisms responsible for these differences remain to be identified. The resistant strain exhibited a more robust cell-mediated immune response, whereas the sensitive strains showed a less effective cell-mediated response, and the infection was associated with signs of demyelination, loss of oligodendrocytes, and increased cytokine signaling. These findings are consistent with our previous observations that STS mice exhibited the highest levels of IL-1 beta mRNA following intracerebral TBEV inoculation compared to other strains. Conversely, most other cytokine/chemokine mRNAs were at their lowest levels in STS mice compared to CcS-11 and/or BALB/c mice. The highest levels of proinflammatory cytokines/chemokines (IFN gamma, CCL3/MIP-1 alpha, CCL2/MCP-1, IP-10) were observed in CcS-11 mice, suggesting that these cytokines/chemokines are associated with a more severe form of TBE in our experimental model. In contrast, STS mice demonstrated the lowest levels of IL-6 and CCL5/RANTES, as well as CCL3/MIP-1 alpha and CCL4/MIP-1 beta mRNAs [6]. This upregulation of cytokines and chemokines in the brain could accelerate the progression of encephalitis rather than promote effective viral restriction [62]. Similar to our study, elevated levels of RANTES, CCL3/MIP-1 alpha, CCL4/MIP-1 beta, and IP-10 mRNA have been associated with a lethal form of West Nile virus infection in mice compared to non-lethal cases [63]. The uncontrolled or excessive release of proinflammatory mediators in the brain can lead to tissue damage, resulting in varying pathologies and disease outcomes [64–66].

Demyelination is a major feature of neuropathology caused by various viruses [67]. In the case of flaviviruses, demyelination and subsequent axonal damage have been observed in mice infected with Japanese encephalitis virus [68]. Conversely, many viruses that infect the nervous system do not cause demyelination. For example, acute infection with rabies virus and lifelong, persistent infection with lymphocytic choriomeningitis virus are not linked with demyelination [67]. Our study suggests that demyelination could also be associated with TBEV infection in mice, with the degree of demyelination potentially being dependent on the host genotype. In monkeys with chronic TBE, destructive changes in oligodendrocytes and demyelination have been described, along with other pathological changes, such as neuronal and astrocyte damage, vascular wall deterioration, severe brain tissue edema with signs of spongiform degeneration, absence of cell proliferation, perivascular cell infiltration, glial nodules, and circulatory disorders [69]. Whether the observed signs of demyelination and loss of oligodendrocytes in more sensitive strains, as revealed by transcriptomic analysis, simply reflect higher levels of pathology driven by other genotype-related responses, including excessive immunopathological reactions in the brain, or whether these mice are inherently more prone to this type of neural damage than the resistant strain remains unknown. Nevertheless, this observation aligns with our other data, which clearly demonstrate an exaggerated immune and immunopathological response, along with increased pathology following TBEV infection in sensitive strains compared to the resistant strain. Importantly, our findings suggest that the severity of pathology does not correlate with virus titer in the brain. Further research into the demyelination phenomenon during TBEV infection of the brain is necessary, as it could have significant implications in understanding the neuropathology and severity of TBE.

### Limitations and future prospects

The present study has several limitations. While mice serve as a reliable model for TBE, as TBEV-infected mice exhibit pathology similar to severe TBE cases in humans, the findings from rodent models are not always directly translatable to human conditions due to well-documented differences in both innate and adaptive immunity between mice and humans. Additionally, our study included only female mice, and as a result, sex-related differences in immune responses to infection were not evaluated. Future studies should aim to address these limitations by incorporating both sexes into experimental designs and exploring additional models to validate findings in a human context. We were also unable to identify specific genetic polymorphisms associated with the distinct immune responses to infection observed in the individual mouse strains. In our previous work, we identified nine potential genes that may be associated with survival following infection. Future studies utilizing knockdown or overexpression approaches, either in vitro or in vivo, could help elucidate the precise roles of these genes during TBEV infection.

## Conclusions

In conclusion, our study underscores the pivotal role of genetic background in shaping susceptibility to TBEV infection and its clinical manifestations. Leveraging mice as models for human TBE, we developed a model that mirrors varying susceptibilities based on host genotype, revealing distinct immune responses and gene expression profiles among different strains. Notably, differences in the expression of candidate genes across tissues suggest unique roles in mediating susceptibility or resistance to the virus, potentially influencing initial responses upon TBEV entry into the host organism. Our findings indicate that, though certain strains exhibit predispositions to the infection and virus-induced pathology, such as impaired antibody production and exaggerated cytokine responses, others may mount more effective antiviral defenses. Notably, macrophages play a critical role in modulating susceptibility, with differing responses observed even prior to infection, underscoring their importance in determining disease outcomes. Transcriptomic analyses of brain tissue following TBEV infection revealed strain-specific responses, implicating immune and inflammatory pathways in disease severity. The observed demyelination and neural damage in sensitive strains highlight potential genotype-related vulnerabilities, suggesting that these mice may be predisposed to more severe neuropathology. Importantly, our study emphasizes that the severity of pathology is not dictated solely by viral load, but also by the host’s immunopathological response. These insights into the genetic determinants of TBEV susceptibility and associated neuropathology will pave the way for further investigation.

## Electronic supplementary material

Below is the link to the electronic supplementary material.

Supplementary Material 1

Supplementary Material 2

## Data Availability

The data were deposited in NCBI’s Gene Expression Omnibus and are accessible through GEO Series Accession number GSE276086.

## References

[1] Ruzek D, Avšič Županc T, Borde J, Chrdle A, Eyer L, Karganova G, Kholodilov I, Knap N, Kozlovskaya L, Matveev A, et al. Tick-borne encephalitis in Europe and Russia: review of pathogenesis, clinical features, therapy, and vaccines. Antiviral Res. 2019;164:23–51. 30710567 10.1016/j.antiviral.2019.01.014

[2] Chiffi G, Grandgirard D, Leib SL, Chrdle A, Růžek D. Tick-borne encephalitis: a comprehensive review of the epidemiology, virology, and clinical picture. Rev Med Virol 2023:e2470. 10.1002/rmv.2470 37392370

[3] Postler TS, Beer M, Blitvich BJ, Bukh J, de Lamballerie X, Drexler JF, Imrie A, Kapoor A, Karganova GG, Lemey P, et al. Renaming of the genus Flavivirus to Orthoflavivirus and extension of binomial species names within the family Flaviviridae. Arch Virol. 2023;168:224. 37561168 10.1007/s00705-023-05835-1

[4] Bogovic P, Lotric-Furlan S, Strle F. What tick-borne encephalitis may look like: clinical signs and symptoms. Travel Med Infect Dis. 2010;8:246–50. 20970727 10.1016/j.tmaid.2010.05.011

[5] Bogovic P, Strle F. Tick-borne encephalitis: a review of epidemiology, clinical characteristics, and management. World J Clin Cases. 2015;3:430–41. 25984517 10.12998/wjcc.v3.i5.430 PMC4419106

[6] Palus M, Vojtíšková J, Salát J, Kopecký J, Grubhoffer L, Lipoldová M, Demant P, Růžek D. Mice with different susceptibility to tick-borne encephalitis virus infection show selective neutralizing antibody response and inflammatory reaction in the central nervous system. J Neuroinflammation. 2013;10:77. 23805778 10.1186/1742-2094-10-77 PMC3700758

[7] Démant P, Hart AA. Recombinant congenic strains–a new tool for analyzing genetic traits determined by more than one gene. Immunogenetics. 1986;24:416–22. 3793154 10.1007/BF00377961

[8] Palus M, Sohrabi Y, Broman KW, Strnad H, Šíma M, Růžek D, Volkova V, Slapničková M, Vojtíšková J, Mrázková L, et al. A novel locus on mouse chromosome 7 that influences survival after infection with tick-borne encephalitis virus. BMC Neurosci. 2018;19:39. 29976152 10.1186/s12868-018-0438-8 PMC6034256

[9] Labuda M, Austyn JM, Zuffova E, Kozuch O, Fuchsberger N, Lysy J, Nuttall PA. Importance of localized skin infection in tick-borne encephalitis virus transmission. Virology. 1996;219:357–66. 8638401 10.1006/viro.1996.0261

[10] Khozinsky VV, Semenov BF, Gresíková M, Chunikhin SP, Sekeyová M, Kozuch O. Role of macrophages in the pathogenesis of experimental tick-borne encephalitis in mice. Acta Virol. 1985;29:194–202. 2864820

[11] Mims CA. THE PERITONEAL MACROPHAGES OF MICE. Br J Exp Pathol. 1964;45:37–43. 14119272 PMC2094654

[12] Mims CA. ASPECTS OF THE PATHOGENESIS OF VIRUS DISEASES. Bacteriol Rev. 1964;28:30–71. 14127970 10.1128/br.28.1.30-71.1964 PMC441209

[13] Murray PJ. Macrophage polarization. Annu Rev Physiol. 2017;79:541–66. 27813830 10.1146/annurev-physiol-022516-034339

[14] Růžek D, Dobler G, Donoso Mantke O. Tick-borne encephalitis: pathogenesis and clinical implications. Travel Med Infect Dis. 2010;8:223–32. 20970725 10.1016/j.tmaid.2010.06.004

[15] Gelpi E, Preusser M, Garzuly F, Holzmann H, Heinz FX, Budka H. Visualization of central European tick-borne encephalitis infection in fatal human cases. J Neuropathol Exp Neurol. 2005;64:506–12. 15977642 10.1093/jnen/64.6.506

[16] Gelpi E, Preusser M, Laggner U, Garzuly F, Holzmann H, Heinz FX, Budka H. Inflammatory response in human tick-borne encephalitis: analysis of postmortem brain tissue. J Neurovirol. 2006;12:322–7. 16966222 10.1080/13550280600848746

[17] De Madrid AT, Porterfield JS. A simple micro-culture method for the study of group B arboviruses. Bull World Health Organ. 1969;40:113–21. 4183812 PMC2554446

[18] Pokorna Formanova P, Palus M, Salat J, Hönig V, Stefanik M, Svoboda P, Ruzek D. Changes in cytokine and chemokine profiles in mouse serum and brain, and in human neural cells, upon tick-borne encephalitis virus infection. J Neuroinflammation. 2019;16:205. 31699097 10.1186/s12974-019-1596-z PMC6839073

[19] Sohrabi Y, Volkova V, Kobets T, Havelková H, Krayem I, Slapničková M, Demant P, Lipoldová M. Genetic regulation of guanylate-binding proteins 2b and 5 during Leishmaniasis in mice. Front Immunol. 2018;9:130. 29467757 10.3389/fimmu.2018.00130 PMC5808352

[20] Krayem I, Sohrabi Y, Javorková E, Volkova V, Strnad H, Havelková H, Vojtíšková J, Aidarova A, Holáň V, Demant P, Lipoldová M. Genetic influence on frequencies of myeloid-derived cell subpopulations in mouse. Front Immunol. 2022;12:760881. 35154069 10.3389/fimmu.2021.760881 PMC8826059

[21] Arvidsson S, Kwasniewski M, Riaño-Pachón DM, Mueller-Roeber B. QuantPrime–a flexible tool for reliable high-throughput primer design for quantitative PCR. BMC Bioinformatics. 2008;9:465. 18976492 10.1186/1471-2105-9-465 PMC2612009

[22] Ritchie ME, Phipson B, Wu D, Hu Y, Law CW, Shi W. Smyth GK: limma powers differential expression analyses for RNA-sequencing and microarray studies. Nucleic Acids Res. 2015;43:e47. 25605792 10.1093/nar/gkv007 PMC4402510

[23] Wu T, Hu E, Xu S, Chen M, Guo P, Dai Z, Feng T, Zhou L, Tang W, Zhan L, et al. clusterProfiler 4.0: a universal enrichment tool for interpreting omics data. Innov (Camb). 2021;2:100141. 10.1016/j.xinn.2021.100141 PMC8454663 34557778

[24] Ashburner M, Ball CA, Blake JA, Botstein D, Butler H, Cherry JM, Davis AP, Dolinski K, Dwight SS, Eppig JT, et al. Gene ontology: tool for the unification of biology. The Gene Ontology Consortium. Nat Genet. 2000;25:25–9. 10802651 10.1038/75556 PMC3037419

[25] Aleksander SA, Balhoff J, Carbon S, Cherry JM, Drabkin HJ, Ebert D, Feuermann M, Gaudet P, Harris NL, Hill DP et al. The Gene Ontology knowledgebase in 2023. Genetics 2023, 224. 10.1093/genetics/iyad031 PMC10158837 36866529

[26] Edgar R, Domrachev M, Lash AE. Gene expression Omnibus: NCBI gene expression and hybridization array data repository. Nucleic Acids Res. 2002;30:207–10. 11752295 10.1093/nar/30.1.207 PMC99122

[27] Crocker PR, Paulson JC, Varki A. Siglecs and their roles in the immune system. Nat Rev Immunol. 2007;7:255–66. 17380156 10.1038/nri2056

[28] Xu X, Masubuchi T, Cai Q, Zhao Y, Hui E. Molecular features underlying differential SHP1/SHP2 binding of immune checkpoint receptors. Elife 2021, 10. 10.7554/eLife.74276 PMC8631942 34734802

[29] Barkhash AV, Babenko VN, Kobzev VF, Romaschenko AG, Voevoda MI. Polymorphism of 2’-5’-oligoadenylate synthetase (OAS) genes, associated with predisposition to severe forms of tick-borne encephalitis, in human populations of North Eurasia. Mol Biol. 2010;44:875–82. 32214471 10.1134/S002689331006004X PMC7088653

[30] Barkhash AV, Babenko VN, Voevoda MI, Romaschenko AG. Association of IL28B and IL10 gene polymorphism with predisposition to tick-borne encephalitis in a Russian population. Ticks Tick Borne Dis. 2016;7:808–12. 27068548 10.1016/j.ttbdis.2016.03.019

[31] Barkhash AV, Perelygin AA, Babenko VN, Brinton MA, Voevoda MI. Single nucleotide polymorphism in the promoter region of the CD209 gene is associated with human predisposition to severe forms of tick-borne encephalitis. Antiviral Res. 2012;93:64–8. 22061615 10.1016/j.antiviral.2011.10.017

[32] Barkhash AV, Perelygin AA, Babenko VN, Myasnikova NG, Pilipenko PI, Romaschenko AG, Voevoda MI, Brinton MA. Variability in the 2’-5’-oligoadenylate synthetase gene cluster is associated with human predisposition to tick-borne encephalitis virus-induced disease. J Infect Dis. 2010;202:1813–8. 21050126 10.1086/657418

[33] Barkhash AV, Voevoda MI, Romaschenko AG. Association of single nucleotide polymorphism rs3775291 in the coding region of the TLR3 gene with predisposition to tick-borne encephalitis in a Russian population. Antiviral Res. 2013;99:136–8. 23721942 10.1016/j.antiviral.2013.05.008

[34] Barkhash AV, Yurchenko AA, Yudin NS, Ignatieva EV, Kozlova IV, Borishchuk IA, Pozdnyakova LL, Voevoda MI, Romaschenko AG. A matrix metalloproteinase 9 (MMP9) gene single nucleotide polymorphism is associated with predisposition to tick-borne encephalitis virus-induced severe central nervous system disease. Ticks Tick Borne Dis. 2018;9:763–7. 29496490 10.1016/j.ttbdis.2018.02.010

[35] Fortova A, Barkhash AV, Pychova M, Krbkova L, Palus M, Salat J, Ruzek D. Genetic polymorphisms in innate immunity genes influence predisposition to tick-borne encephalitis. J Neurovirol. 2023;29:699–705. 37898570 10.1007/s13365-023-01182-8 PMC10794283

[36] Fortova A, Hönig V, Salat J, Palus M, Pychova M, Krbkova L, Barkhash AV, Kriha MF, Chrdle A, Lipoldova M, Ruzek D. Serum matrix metalloproteinase-9 (MMP-9) as a biomarker in paediatric and adult tick-borne encephalitis patients. Virus Res. 2023;324:199020. 36528170 10.1016/j.virusres.2022.199020 PMC10194185

[37] Ignatieva EV, Yurchenko AA, Voevoda MI, Yudin NS. Exome-wide search and functional annotation of genes associated in patients with severe tick-borne encephalitis in a Russian population. BMC Med Genomics. 2019;12:61. 31122248 10.1186/s12920-019-0503-x PMC6533173

[38] Czupryna P, Parczewski M, Grygorczuk S, Pancewicz S, Zajkowska J, Dunaj J, Kondrusik M, Krawczuk K, Moniuszko-Malinowska A. Analysis of the relationship between single nucleotide polymorphism of the CD209, IL-10, IL-28 and CCR5 D32 genes with the human predisposition to developing tick-borne encephalitis. Postepy Hig Med Dosw (Online). 2017;71:788–96. 28894041 10.5604/01.3001.0010.3856

[39] Mickienė A, Pakalnienė J, Nordgren J, Carlsson B, Hagbom M, Svensson L, Lindquist L. Polymorphisms in chemokine receptor 5 and toll-like receptor 3 genes are risk factors for clinical tick-borne encephalitis in the Lithuanian population. PLoS ONE. 2014;9:e106798. 25226020 10.1371/journal.pone.0106798 PMC4165893

[40] Kindberg E, Mickiene A, Ax C, Akerlind B, Vene S, Lindquist L, Lundkvist A, Svensson L. A deletion in the chemokine receptor 5 (CCR5) gene is associated with tickborne encephalitis. J Infect Dis. 2008;197:266–9. 18179389 10.1086/524709

[41] Mandl CW. Steps of the tick-borne encephalitis virus replication cycle that affect neuropathogenesis. Virus Res. 2005;111:161–74. 15871909 10.1016/j.virusres.2005.04.007

[42] Kobets T, Čepičková M, Volkova V, Sohrabi Y, Havelková H, Svobodová M, Demant P, Lipoldová M. Novel loci Controlling Parasite load in organs of mice infected with Leishmania major, their interactions and sex influence. Front Immunol. 2019;10:1083. 31231359 10.3389/fimmu.2019.01083 PMC6566641

[43] Kobets T, Havelková H, Grekov I, Volkova V, Vojtíšková J, Slapničková M, Kurey I, Sohrabi Y, Svobodová M, Demant P, Lipoldová M. Genetics of host response to Leishmania Tropica in mice - different control of skin pathology, chemokine reaction, and invasion into spleen and liver. PLoS Negl Trop Dis. 2012;6:e1667. 22679519 10.1371/journal.pntd.0001667 PMC3367980

[44] Krayem I, Sohrabi Y, Havelková H, Gusareva ES, Strnad H, Čepičková M, Volkova V, Kurey I, Vojtíšková J, Svobodová M, et al. Functionally distinct regions of the locus Leishmania major response 15 control IgE or IFNγ level in addition to skin lesions. Front Immunol. 2023;14:1145269. 37600780 10.3389/fimmu.2023.1145269 PMC10437074

[45] Lipoldová M, Demant P. Gene-Specific Sex effects on susceptibility to Infectious diseases. Front Immunol. 2021;12:712688. 34721380 10.3389/fimmu.2021.712688 PMC8553003

[46] Lipoldová M, Havelková H, Badalova J, Vojtísková J, Quan L, Krulova M, Sohrabi Y, Stassen AP, Demant P. Loci controlling lymphocyte production of interferon c after alloantigen stimulation in vitro and their co-localization with genes controlling lymphocyte infiltration of tumors and tumor susceptibility. Cancer Immunol Immunother. 2010;59:203–13. 19655140 10.1007/s00262-009-0739-y PMC2776939

[47] Mrázek J, Mrázková L, Mekadim C, Jarošíková T, Krayem I, Sohrabi Y, Demant P, Lipoldová M. Effects of Leishmania major infection on the gut microbiome of resistant and susceptible mice. Appl Microbiol Biotechnol. 2024;108:145. 38240984 10.1007/s00253-024-13002-y PMC10799115

[48] Síma M, Havelková H, Quan L, Svobodová M, Jarošíková T, Vojtíšková J, Stassen AP, Demant P, Lipoldová M. Genetic control of resistance to Trypanosoma Brucei brucei infection in mice. PLoS Negl Trop Dis. 2011;5:e1173. 21666791 10.1371/journal.pntd.0001173 PMC3110168

[49] Slapničková M, Volkova V, Čepičková M, Kobets T, Šíma M, Svobodová M, Demant P, Lipoldová M. Gene-specific sex effects on eosinophil infiltration in leishmaniasis. Biol Sex Differ. 2016;7:59. 27895891 10.1186/s13293-016-0117-3 PMC5120444

[50] Sohrabi Y, Havelková H, Kobets T, Šíma M, Volkova V, Grekov I, Jarošíková T, Kurey I, Vojtíšková J, Svobodová M, et al. Mapping the genes for susceptibility and response to Leishmania Tropica in mouse. PLoS Negl Trop Dis. 2013;7:e2282. 23875032 10.1371/journal.pntd.0002282 PMC3708836

[51] Ahantarig A, Růzek D, Vancová M, Janowitz A, St’astná H, Tesarová M, Grubhoffer L. Tick-borne encephalitis virus infection of cultured mouse macrophages. Intervirology. 2009;52:283–90. 19707021 10.1159/000235741

[52] Růzek D, Gritsun TS, Forrester NL, Gould EA, Kopecký J, Golovchenko M, Rudenko N, Grubhoffer L. Mutations in the NS2B and NS3 genes affect mouse neuroinvasiveness of a western European field strain of tick-borne encephalitis virus. Virology. 2008;374:249–55. 18339416 10.1016/j.virol.2008.01.010

[53] Rusanov AL, Stepanov AA, Zgoda VG, Kaysheva AL, Selinger M, Maskova H, Loginov D, Sterba J, Grubhoffer L, Luzgina NG. Proteome dataset of mouse macrophage cell line infected with tick-borne encephalitis virus. Data Brief. 2020;28:105029. 31909125 10.1016/j.dib.2019.105029 PMC6939094

[54] Rusanov AL, Kozhin PM, Tikhonova OV, Zgoda VG, Loginov DS, Chlastáková A, Selinger M, Sterba J, Grubhoffer L, Luzgina NG. Proteome Profiling of PMJ2-R and primary peritoneal macrophages. Int J Mol Sci 2021, 22. 10.3390/ijms22126323 PMC8231560 34204832

[55] Clarke P, Leser JS, Bowen RA, Tyler KL. Virus-induced transcriptional changes in the brain include the differential expression of genes associated with interferon, apoptosis, interleukin 17 receptor A, and glutamate signaling as well as flavivirus-specific upregulation of tRNA synthetases. mBio. 2014;5:e00902–00914. 24618253 10.1128/mBio.00902-14 PMC3952157

[56] Kusuhara H, Sugiyama Y. Active efflux across the blood-brain barrier: role of the solute carrier family. NeuroRx. 2005;2:73–85. 15717059 10.1602/neurorx.2.1.73 PMC539323

[57] Gorman MJ, Poddar S, Farzan M, Diamond MS. The Interferon-stimulated gene Ifitm3 restricts West Nile Virus infection and Pathogenesis. J Virol. 2016;90:8212–25. 27384652 10.1128/JVI.00581-16 PMC5008082

[58] Chmielewska AM, Gómez-Herranz M, Gach P, Nekulova M, Bagnucka MA, Lipińska AD, Rychłowski M, Hoffmann W, Król E, Vojtesek B, et al. The role of IFITM proteins in Tick-Borne Encephalitis Virus infection. J Virol. 2022;96:e0113021. 34613785 10.1128/JVI.01130-21 PMC8754218

[59] Panayiotou C, Lindqvist R, Kurhade C, Vonderstein K, Pasto J, Edlund K, Upadhyay AS, Överby AK. Viperin restricts Zika Virus and Tick-Borne Encephalitis Virus replication by targeting NS3 for proteasomal degradation. J Virol 2018, 92. 10.1128/JVI.02054-17 PMC5972904 29321318

[60] Schoggins JW. Recent advances in antiviral interferon-stimulated gene biology. F1000Res. 2018;7:309. 29568506 10.12688/f1000research.12450.1 PMC5850085

[61] Banus HA, van Kranen HJ, Mooi FR, Hoebee B, Nagelkerke NJ, Demant P, Kimman TG. Genetic control of Bordetella pertussis infection: identification of susceptibility loci using recombinant congenic strains of mice. Infect Immun. 2005;73:741–7. 15664912 10.1128/IAI.73.2.741-747.2005 PMC547026

[62] Biswas SM, Kar S, Singh R, Chakraborty D, Vipat V, Raut CG, Mishra AC, Gore MM, Ghosh D. Immunomodulatory cytokines determine the outcome of Japanese encephalitis virus infection in mice. J Med Virol. 2010;82:304–10. 20029807 10.1002/jmv.21688

[63] Shirato K, Kimura T, Mizutani T, Kariwa H, Takashima I. Different chemokine expression in lethal and non-lethal murine West Nile virus infection. J Med Virol. 2004;74:507–13. 15368509 10.1002/jmv.20205

[64] Rossini G, Landini MP, Gelsomino F, Sambri V, Varani S. Innate host responses to West Nile virus: implications for central nervous system immunopathology. World J Virol. 2013;2:49–56. 24175229 10.5501/wjv.v2.i2.49 PMC3785052

[65] Quaresma JA, Pagliari C, Medeiros DB, Duarte MI, Vasconcelos PF. Immunity and immune response, pathology and pathologic changes: progress and challenges in the immunopathology of yellow fever. Rev Med Virol. 2013;23:305–18. 23873723 10.1002/rmv.1752

[66] King NJ, Getts DR, Getts MT, Rana S, Shrestha B, Kesson AM. Immunopathology of flavivirus infections. Immunol Cell Biol. 2007;85:33–42. 17146465 10.1038/sj.icb.7100012

[67] Fazakerley JK, Walker R. Virus demyelination. J Neurovirol. 2003;9:148–64. 12707846 10.1080/13550280390194046 PMC7095111

[68] Yang H, Wang X, Wang Z, Wang G, Fu S, Li F, Yang L, Yuan Y, Shen K, Wang H, Wang Z. Peripheral nerve Injury Induced by Japanese Encephalitis Virus in C57BL/6 mouse. J Virol. 2023;97:e0165822. 37071015 10.1128/jvi.01658-22 PMC10231255

[69] Erman BA, Tulakina LG, Zubenko AV, Subbotina LS. [Ultrastructural changes in the CNS of monkeys with the chronic form of tick-borne encephalitis]. Arkh Patol. 1985;47:46–52. 4004577

