# Supplementary material for: Genotype-driven sensitivity of mice to tick-borne encephalitis virus correlates with differential host responses in peripheral macrophages and brain
Source: J Neuroinflammation. 2025 Jan 28;22:22. doi: 10.1186/s12974-025-03354-1 (PMC11776336; doi:10.1186/s12974-025-03354-1)
Supplement: Supplementary file 2 — Supplementary Material 2 [file 12974_2025_3354_MOESM2_ESM.pdf]

Supplementary Figures

Genotype-Driven Sensitivity of Mice to Tick-Borne Encephalitis Virus Correlates with Differential Host Responses in Peripheral Macrophages and Brain

Michaela Berankova,[1,2,3#] Jiri Holoubek,[1,2,3#] Vaclav Hönig,[2,3,#] Zuzana Matusova,[4,5#] Martin Palus,[2,3,#] Jiri Salat,[2,3] Imtissal Krayem,[6] Jarmila Vojtiskova,[6] Pavel Svoboda,[3,$] Veronika Pranclova,[2,7] Lukas Valihrach,[4,8] Peter Demant,[9] Marie Lipoldova,[6,10] & Daniel Ruzek[1,2,3,✉]

(1) Department of Experimental Biology, Faculty of Science, Masaryk University, Brno, Czech Republic.

(2) Laboratory of Arbovirology, Institute of Parasitology, Biology Centre of the Czech Academy of Sciences, Ceske Budejovice, Czech Republic.

(3) Laboratory of Emerging Viral Diseases, Veterinary Research Institute, Brno, Czech Republic.

(4) Laboratory of Gene Expression, Institute of Biotechnology of the Czech Academy of Sciences, Vestec, Czech Republic.

(5) Faculty of Science, Charles University, Prague, Czech Republic.

(6) Laboratory of Molecular and Cellular Immunology, Institute of Molecular Genetics, Czech Academy of Sciences, Prague, Czech Republic.

(7) Faculty of Science, University of South Bohemia, Ceske Budejovice, Czech Republic

(8) Department of Cellular Neurophysiology, Institute of Experimental Medicine of the Czech Academy of Sciences, Prague, Czech Republic.

(9) Department of Molecular and Cellular Biology, Roswell Park Comprehensive Cancer Center, Buffalo, NY, USA

(10) Department of Medical Genetics, 3rd Faculty of Medicine, Charles University, Prague, Czech Republic.

# Contributed equally (sorted in alphabetical order)

$ Present Address: Institute of Organic Chemistry and Biochemistry, Czech Academy of Sciences, Prague, Czech Republic.

✉Author for Correspondence; ruzekd@paru.cas.cz

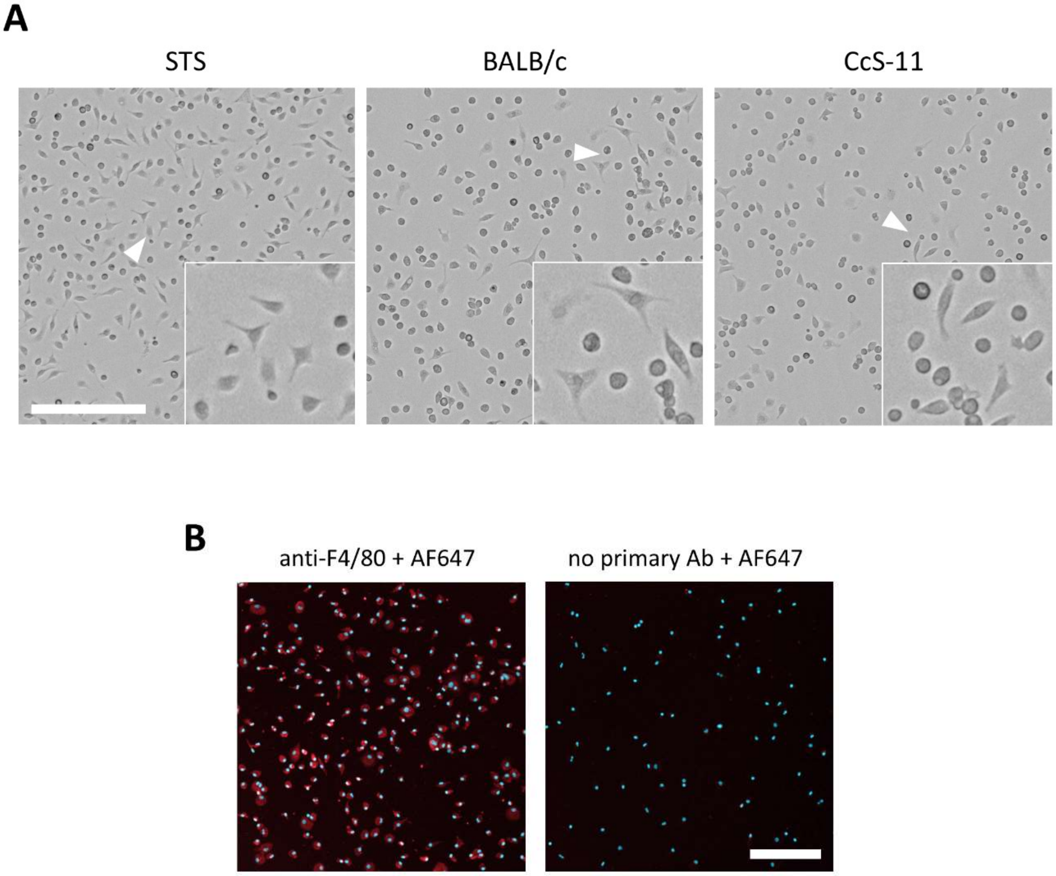

Supplementary Figure 1 | (A) Morphology of the peritoneal macrophages isolated from STS, BALB/c, and CcS-11 mice. (B) The purity of the macrophages was verified using rabbit antibody specific for F4/80, a marker of macrophages. The goat anti-rabbit antibody conjugated with Alexa Fluor 647 was used as a secondary antibody (red) and nuclei were stained with DAPI (blue). Scale bars = 100 µm.

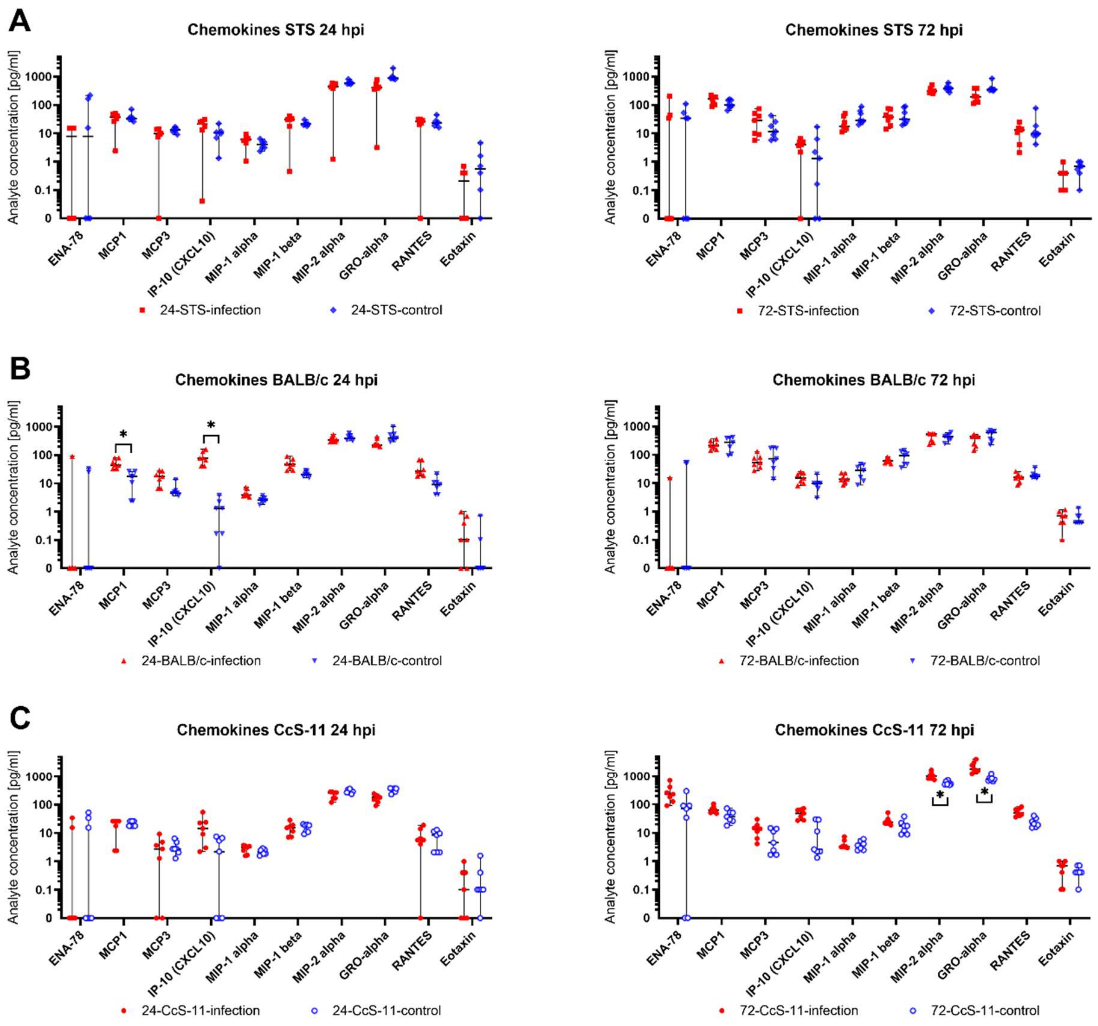

Supplementary Figure 2 | Concentrations of chemokines (median, 95% CI, logarithmic scale) in supernatants of **TBEV infected and uninfected (control) STS, BALB/c, and CcS-11** mouse strain-derived macrophage cultures. Concentrations were determined in five biological replicates for STS and six replicates for CcS-11 and BALB/c derived cultures using Luminex MagPix technology. Statistically significant differences based on the Mann-Whitney multiple comparison test with two-stage step-up multiple comparison corrections (Benjamini, Krieger, Yekutieli; Q=1%) are indicated by asterisks. **(A)** STS-derived macrophages, **(B)** BALB/c-derived macrophages, **(C)** CcS-11-derived macrophages; concentrations 24 hours post-infection on the left and 48 hours on the right.

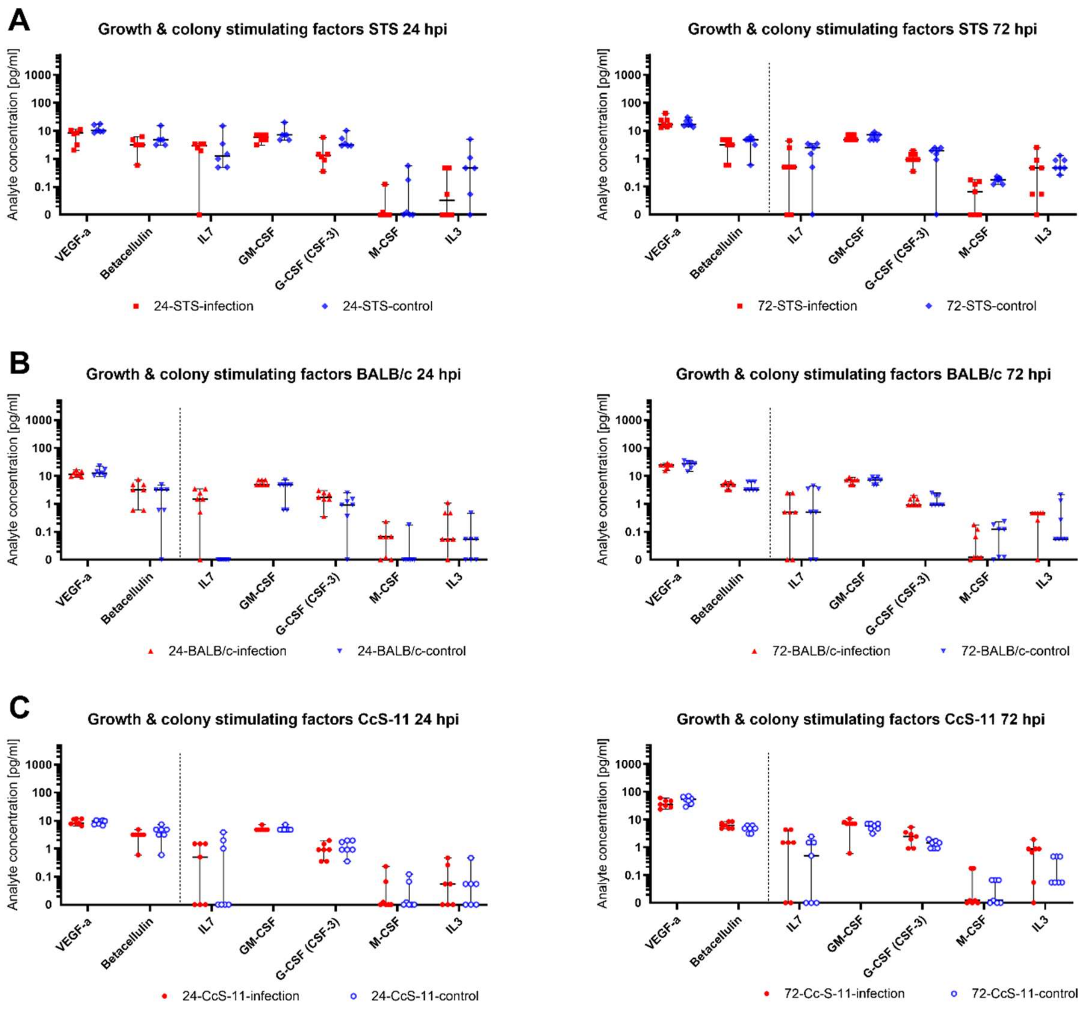

Supplementary Figure 3 | Concentrations of growth and colony-stimulating factors (median, 95% CI, logarithmic scale) in supernatants of **TBEV infected and uninfected (control) STS, BALB/c, and CcS-11** mouse strain-derived macrophage cultures. Concentrations were determined in five biological replicates for STS and six replicates for CcS-11 and BALB/c derived cultures using Luminex MagPix technology. Statistically significant differences based on the Mann-Whitney multiple comparison test with two-stage step-up multiple comparison corrections (Benjamini, Krieger, Yekutieli; Q=1%) are indicated by asterisks. **(A)** STS-derived macrophages, **(B)** BALB/c-derived macrophages, **(C)** CcS-11-derived macrophages; concentrations 24 hours post-infection on the left and 48 hours on the right.

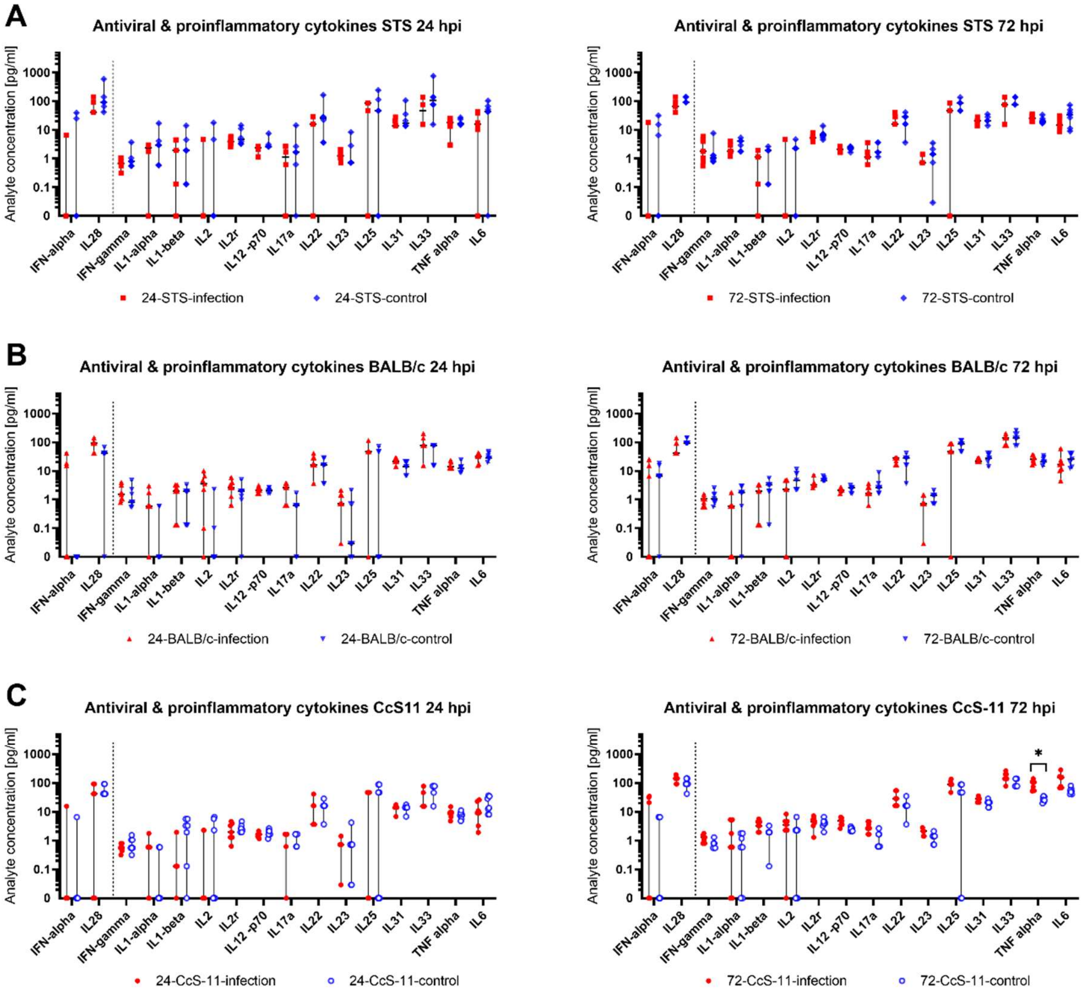

Supplementary Figure 4 | Concentrations of antiviral and proinflammatory cytokines (median, 95% CI, logarithmic scale) in supernatants of **TBEV infected and uninfected (control) STS, BALB/c, and CcS-11** mouse strain-derived macrophage cultures. Concentrations were determined in five biological replicates for STS and six replicates for CcS-11 and BALB/c derived cultures using Luminex MagPix technology. Statistically significant differences based on the Mann-Whitney multiple comparison test with two-stage step-up multiple comparison corrections (Benjamini, Krieger, Yekutieli; Q=1%) are indicated by asterisks. **(A)** STS-derived macrophages, **(B)** BALB/c-derived macrophages, **(C)** CcS-11-derived macrophages; concentrations 24 hours post-infection on the left and 48 hours on the right.

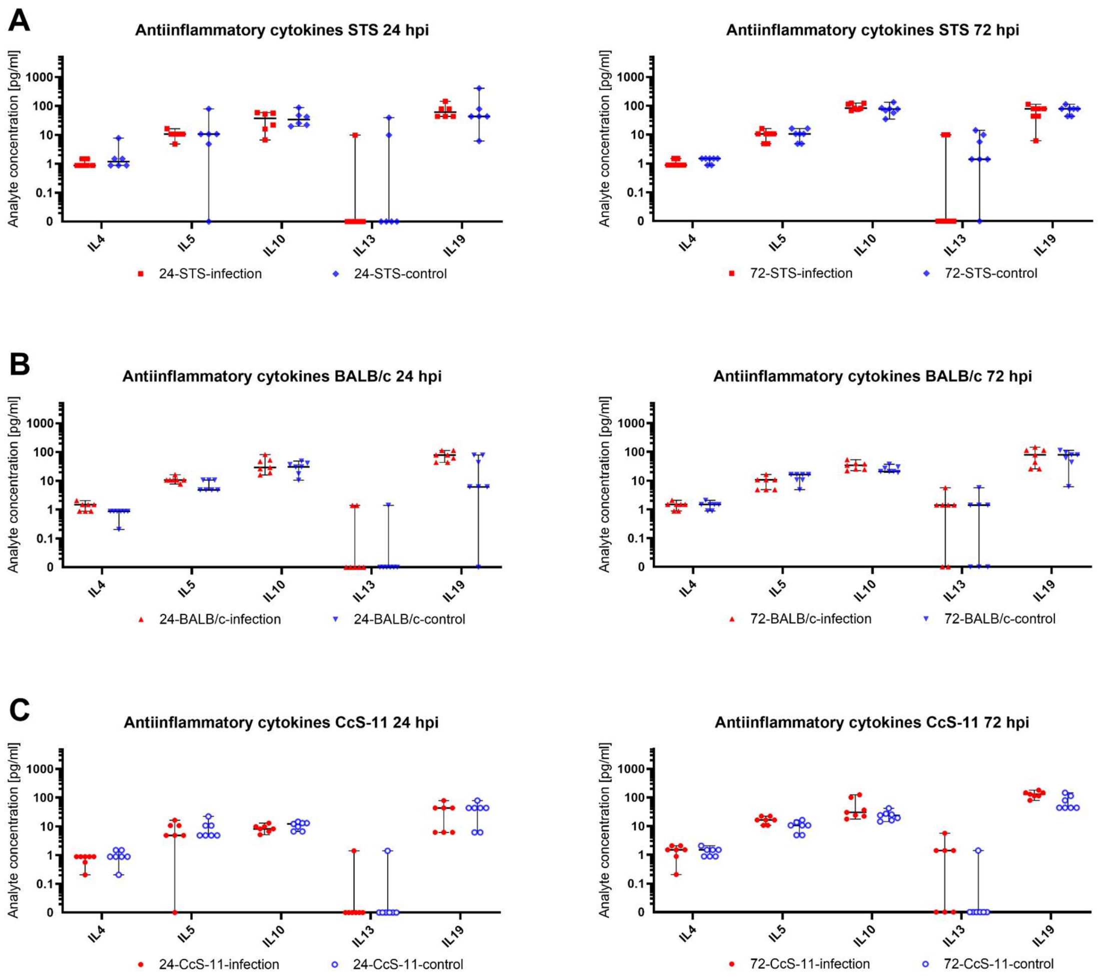

Supplementary Figure 5 | Concentrations of antiinflammatory cytokines (median, 95% CI, logarithmic scale) in supernatants of **TBEV infected and uninfected (control) STS, BALB/c, and CcS-11** mouse strain-derived macrophage cultures. Concentrations were determined in five biological replicates for STS and six replicates for CcS-11 and BALB/c derived cultures using Luminex MagPix technology. Statistically significant differences based on the Mann-Whitney multiple comparison test with two-stage step-up multiple comparison corrections (Benjamini, Krieger, Yekutieli; Q=1%) are indicated by asterisks. **(A)** STS-derived macrophages, **(B)** BALB/c-derived macrophages, **(C)** CcS-11-derived macrophages; concentrations 24 hours post-infection on the left and 48 hours on the right.

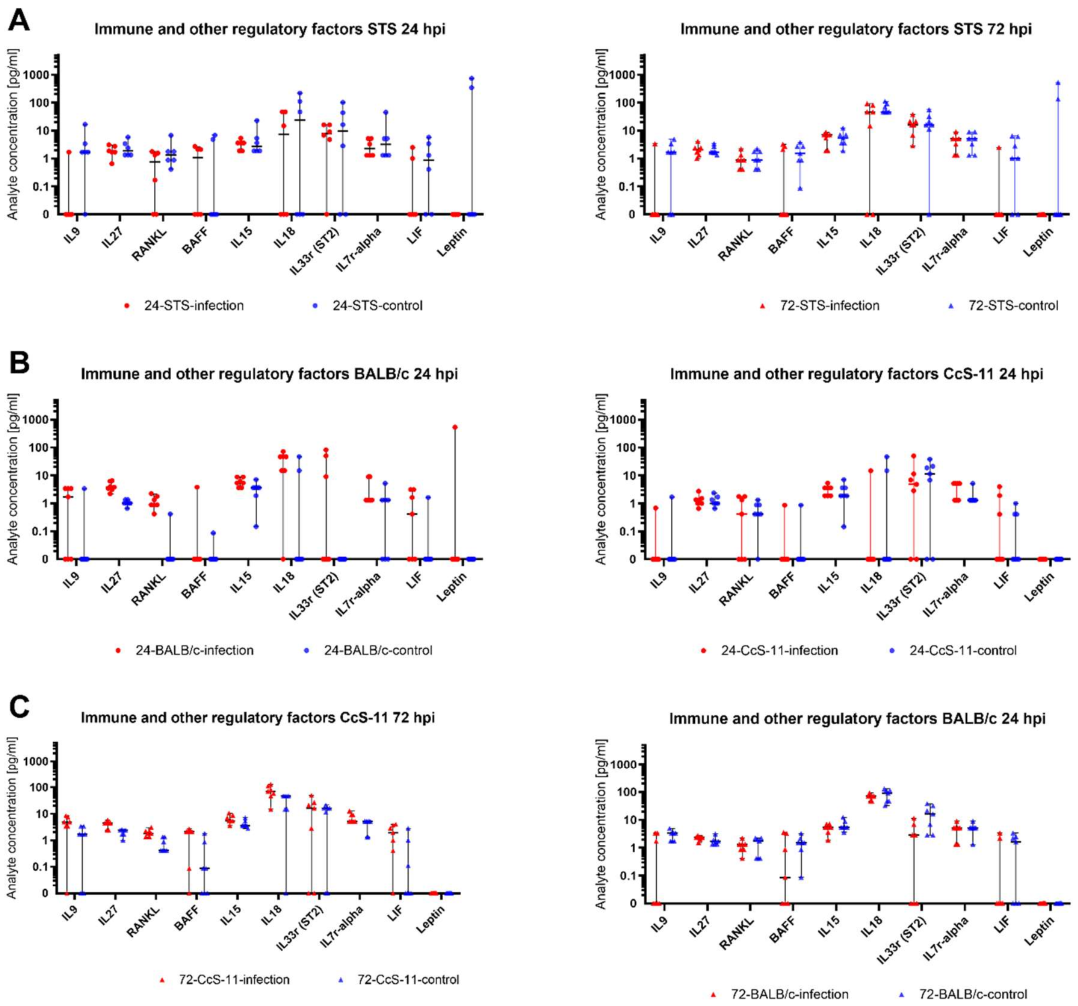

Supplementary Figure 6 | Concentrations of immune and other regulatory factors (median, 95% CI, logarithmic scale) in supernatants of **TBEV infected and uninfected (control) STS, BALB/c, and CcS-11** mouse strain-derived macrophage cultures. Concentrations were determined in five biological replicates for STS and six replicates for CcS-11 and BALB/c derived cultures using Luminex MagPix technology. Statistically significant differences based on the Mann-Whitney multiple comparison test with two-stage step-up multiple comparison corrections (Benjamini, Krieger, Yekutieli; Q=1%) are indicated by asterisks. **(A)** STS-derived macrophages, **(B)** BALB/c-derived macrophages, **(C)** CcS-11-derived macrophages; concentrations 24 hours post-infection on the left and 48 hours on the right.

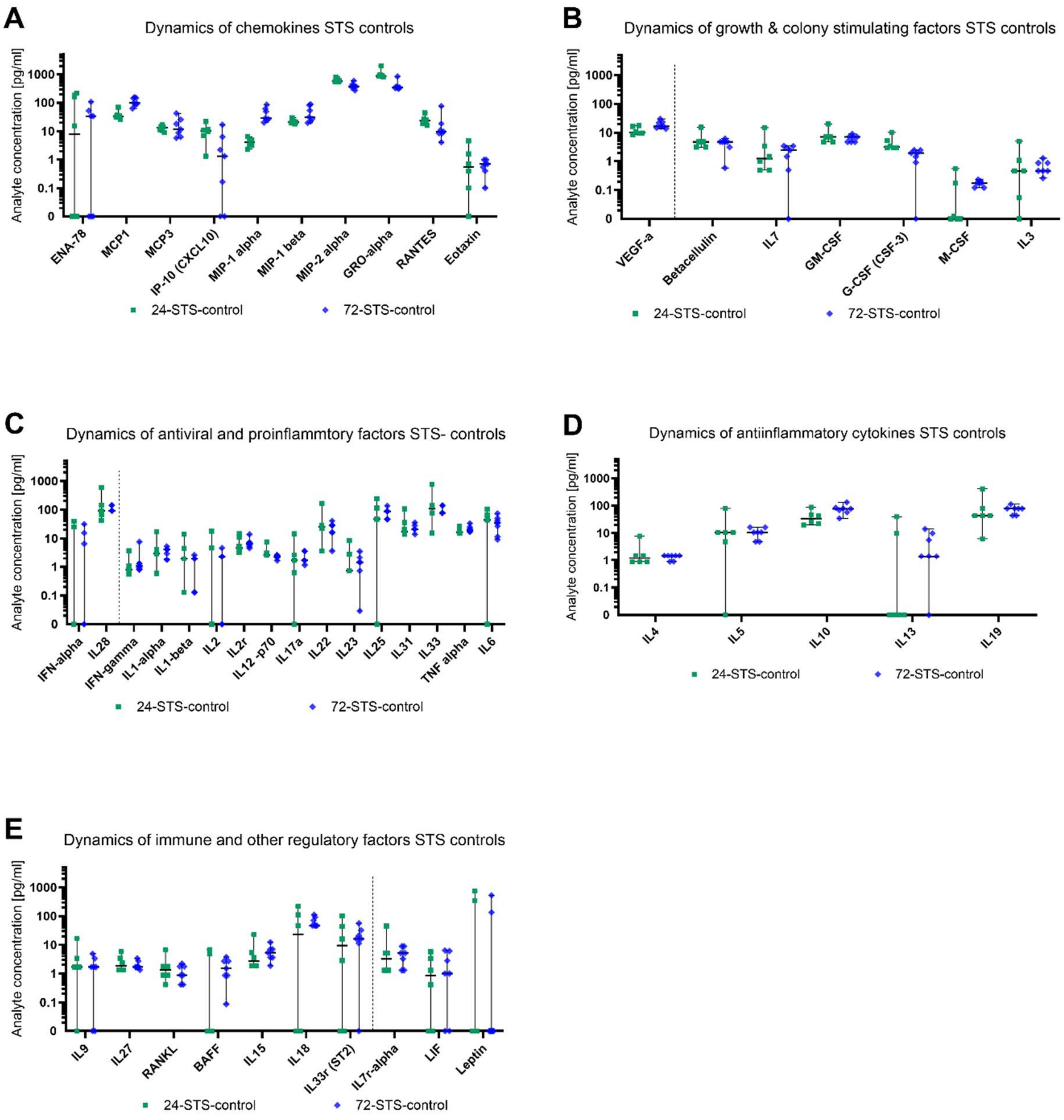

Supplementary Figure 7 | Dynamics (comparison after 24 and 72 hours of cultivation) **of analyte concentrations** (median, 95% CI, logarithmic scale) in supernatants of **uninfected (control) STS mouse strain-derived macrophage cultures**. Concentrations were determined in five biological replicates using Luminex MagPix technology. Statistically significant differences based on the Mann-Whitney multiple comparison test with two-stage step-up multiple comparison corrections (Benjamini, Krieger, Yekutieli; Q=1%) are indicated by asterisks. **(A)** chemokines, **(B)** growth and colony stimulating factors, **(C)** antiviral and proinflammatory factors, **(D)** antiinflammatory cytokines, **(E)** immune and other regulatory factors.

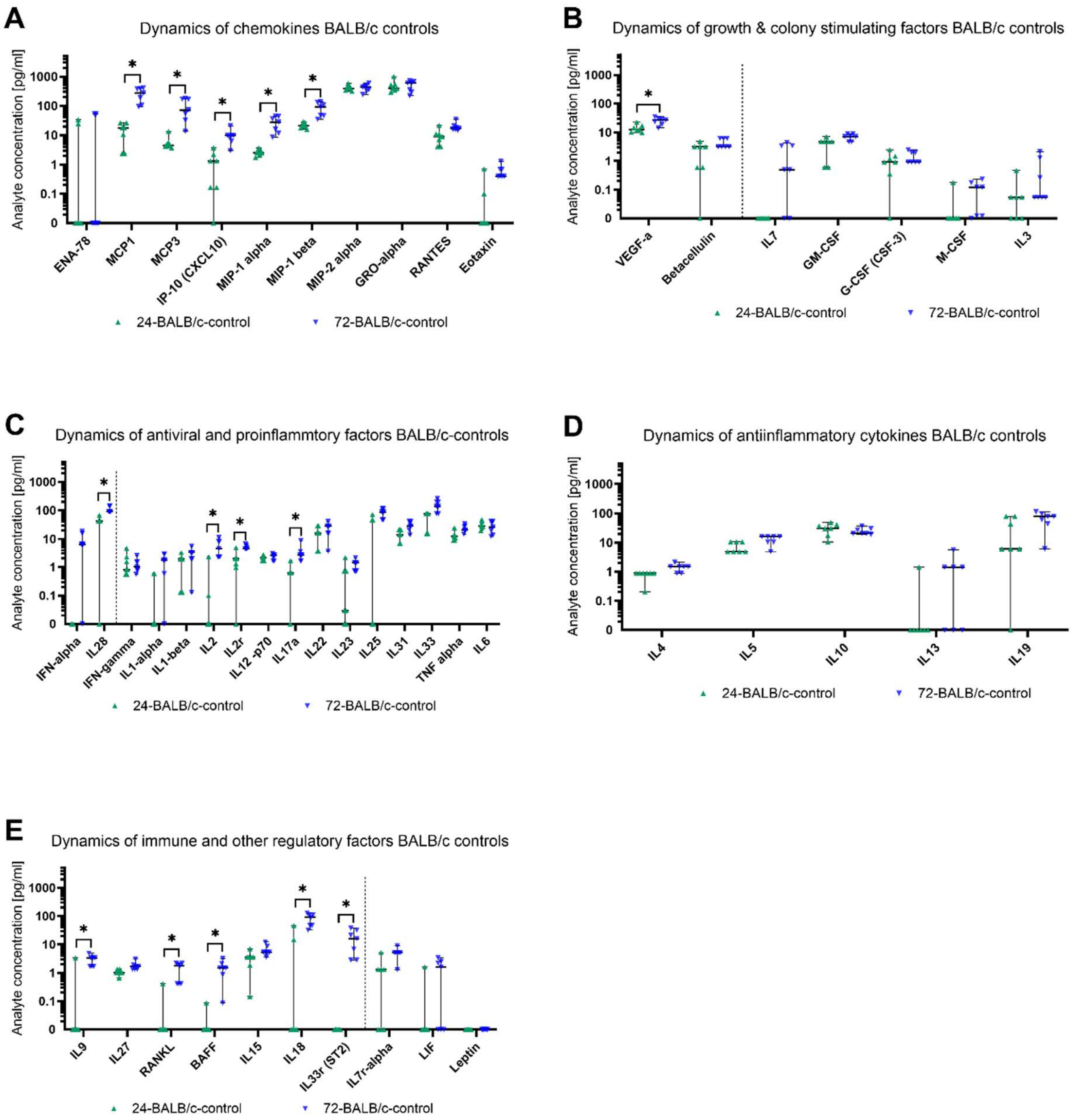

Supplementary Figure 8 | Dynamics (comparison after 24 and 72 hours of cultivation) **of analyte concentrations** (median, 95% CI, logarithmic scale) in supernatants of **uninfected (control) BALB/c mouse strain-derived macrophage cultures**. Concentrations were determined in six biological replicates using Luminex MagPix technology. Statistically significant differences based on the Mann-Whitney multiple comparison test with two-stage step-up multiple comparison corrections (Benjamini, Krieger, Yekutieli; Q=1%) are indicated by asterisks. **(A)** chemokines, **(B)** growth and colony stimulating factors, **(C)** antiviral and proinflammatory factors, **(D)** antiinflammatory cytokines, **(E)** immune and other regulatory factors.

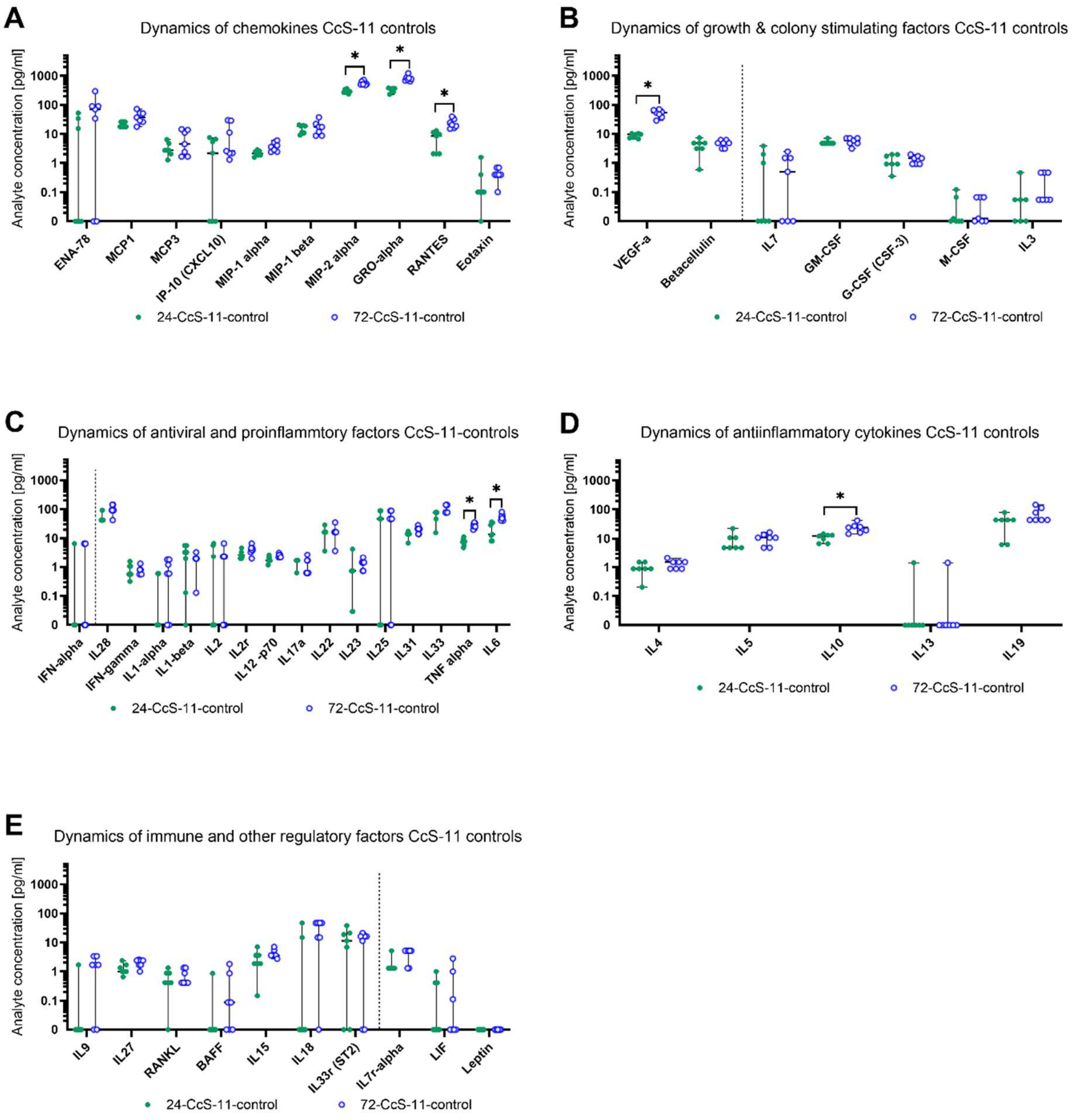

Supplementary Figure 9 | Dynamics (comparison after 24 and 72 hours of cultivation) **of analyte concentrations** (median, 95% CI, logarithmic scale) in supernatants of **uninfected (control) CcS-11 mouse strain-derived macrophage cultures**. Concentrations were determined in six biological replicates using Luminex MagPix technology. Statistically significant differences based on the Mann-Whitney multiple comparison test with two-stage step-up multiple comparison corrections (Benjamini, Krieger, Yekutieli; Q=1%) are indicated by asterisks. **(A)** chemokines, **(B)** growth and colony stimulating factors, **(C)** antiviral and proinflammatory factors, **(D)** antiinflammatory cytokines, **(E)** immune and other regulatory factors.

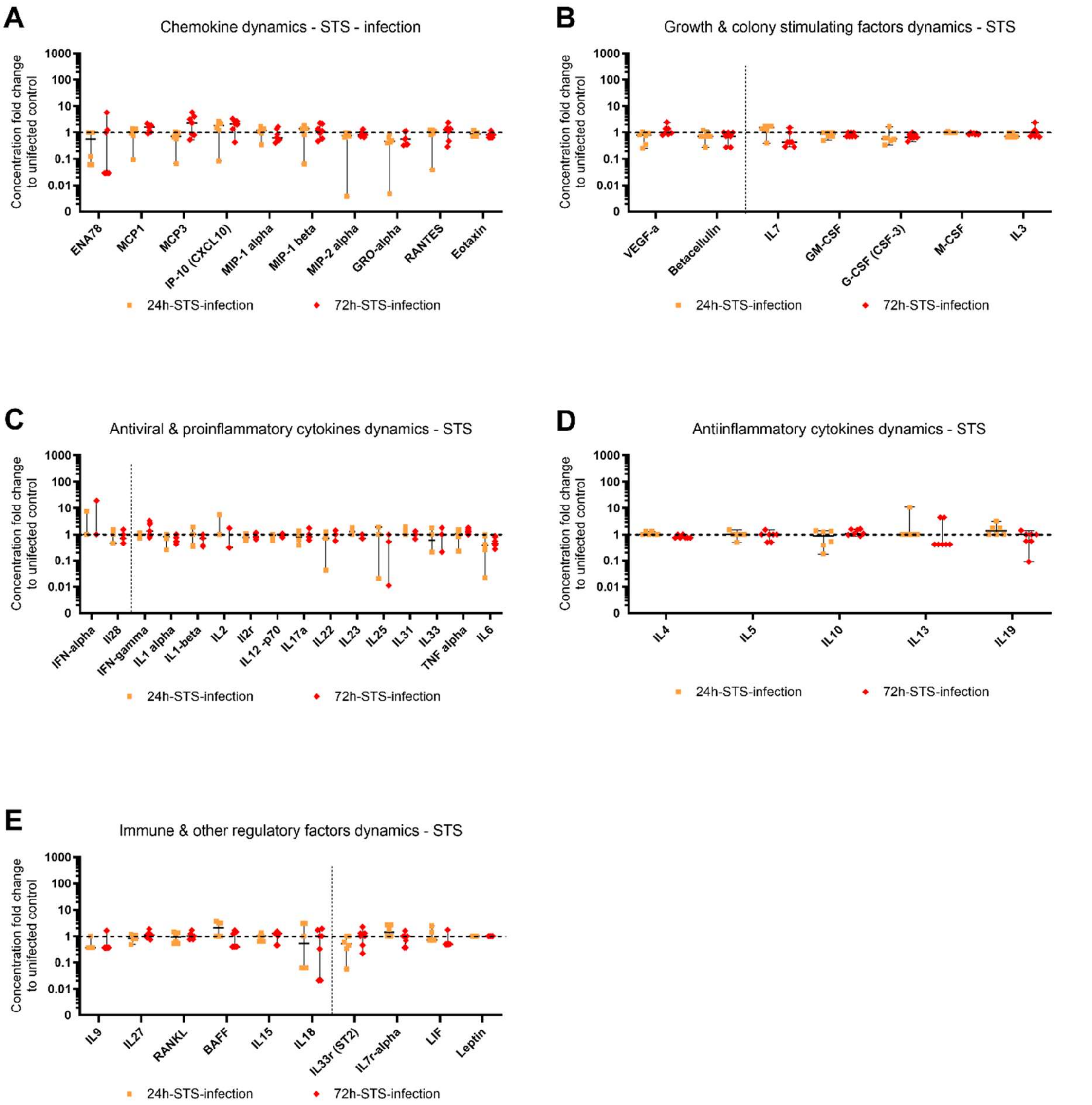

Supplementary Figure 10 | Analyte concentration dynamics in TBEV infected STS mouse strain-derived macrophage cultures presented as concentration fold change to uninfected control (median, 95% CI, logarithmic scale) 24 and 72 hours after TBEV infection. Concentrations were determined in five biological replicates using the Luminex MagPix technology. Statistically significant differences (24 to 72 h), based on multiple Mann-Whitney test with two-stage step-up multiple comparison corrections (Benjamini, Krieger, Yekutieli; Q=1%) are indicated by an asterisk. The horizontal dashed line indicates the concentration level in non-infected control. **(A)** chemokines, **(B)** growth and colony stimulating factors, **(C)** antiviral and proinflammatory factors, **(D)** antiinflammatory cytokines, **(E)** immune and other regulatory factors.

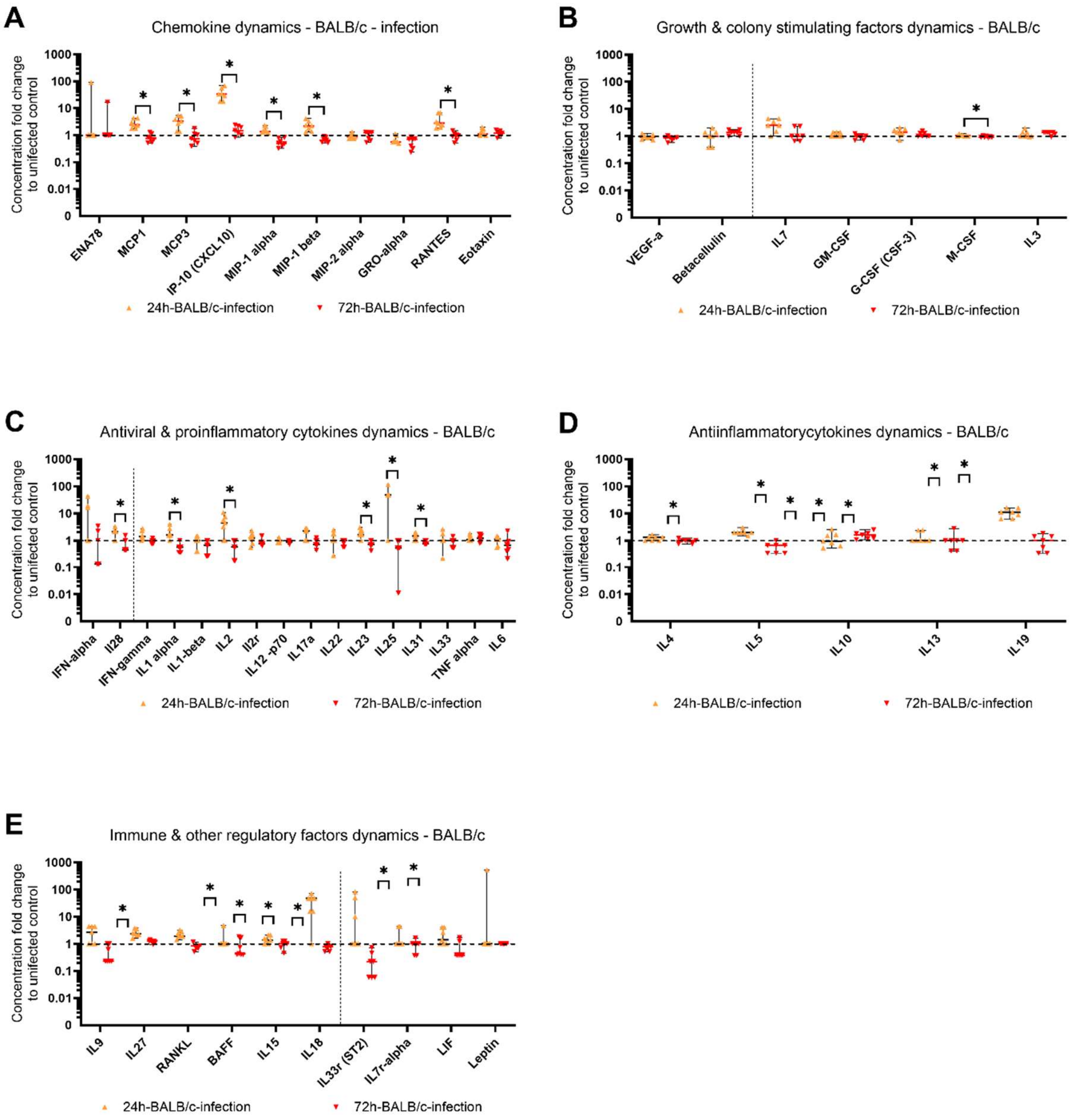

Supplementary Figure 11 | Analyte concentration dynamics in TBEV infected BALB/c mouse strain-derived macrophage cultures presented as concentration fold change to uninfected control (median, 95% CI, logarithmic scale) 24 and 72 hours after TBEV infection. Concentrations were determined in six biological replicates using the Luminex MagPix technology. Statistically significant differences (24 to 72 h), based on multiple Mann-Whitney test with two-stage step-up multiple comparison correction (Benjamini, Krieger, Yekutieli; Q=1%) are indicated by an asterisk. The horizontal dashed line indicates the concentration level in non-infected control. **(A)** chemokines, **(B)** growth and colony stimulating factors, **(C)** antiviral and proinflammatory factors, **(D)** antiinflammatory cytokines, **(E)** immune and other regulatory factors.

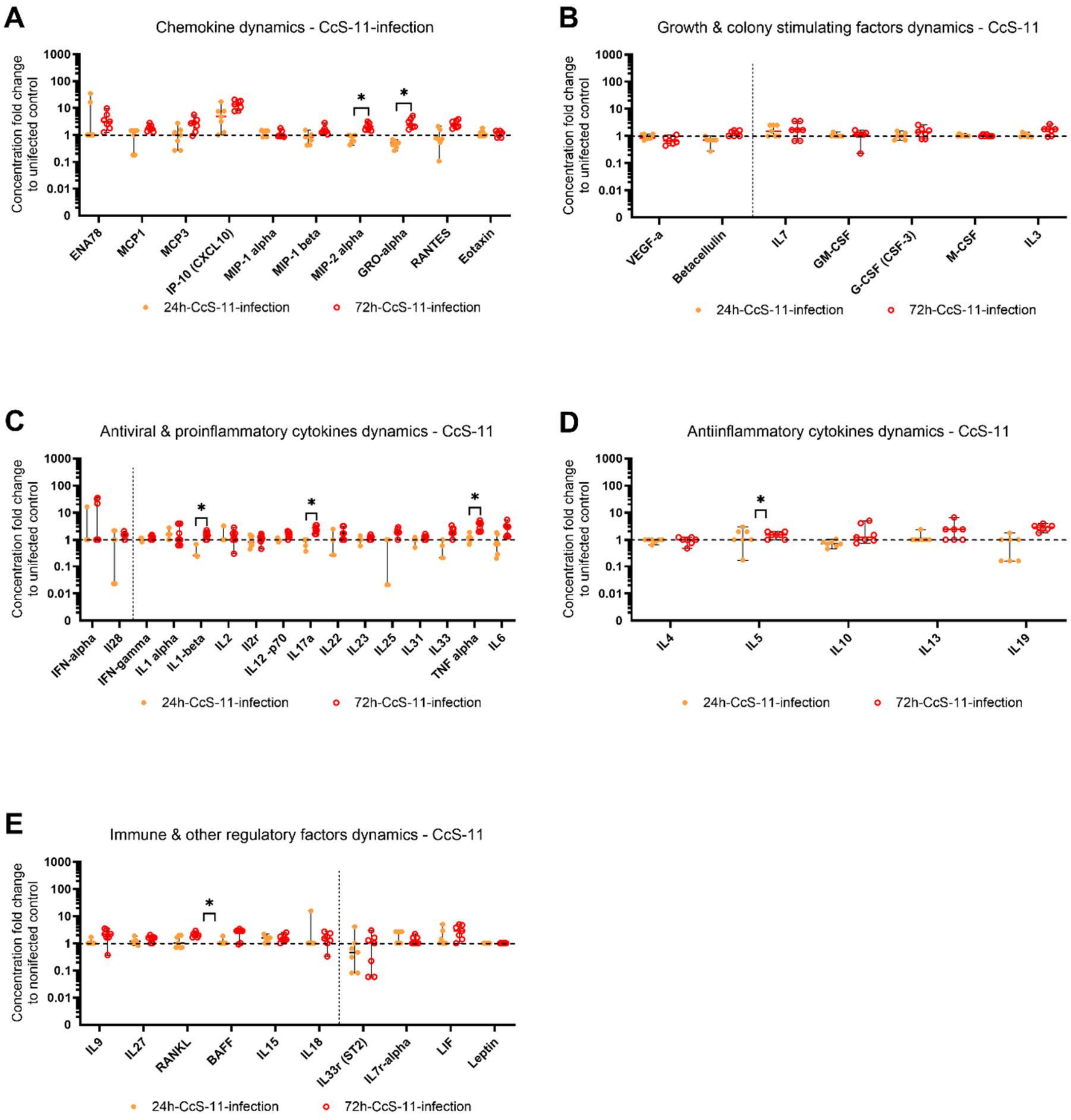

Supplementary Figure 12 | Analyte concentration dynamics in TBEV infected CCS-11 mouse strain-derived macrophage cultures presented as concentration fold change to uninfected control (median, 95% CI, logarithmic scale) 24 and 72 hours after TBEV infection. Concentrations were determined in six biological replicates using the Luminex MagPix technology. Statistically significant differences (24 to 72 h), based on multiple Mann-Whitney test with two-stage step-up multiple comparison corrections (Benjamini, Krieger, Yekutieli; Q=1%) are indicated by an asterisk. The horizontal dashed line indicates the concentration level in non-infected control. **(A)** chemokines, **(B)** growth and colony-stimulating factors, **(C)** antiviral and proinflammatory factors, **(D)** antiinflammatory cytokines, **(E)** immune and other regulatory factors.

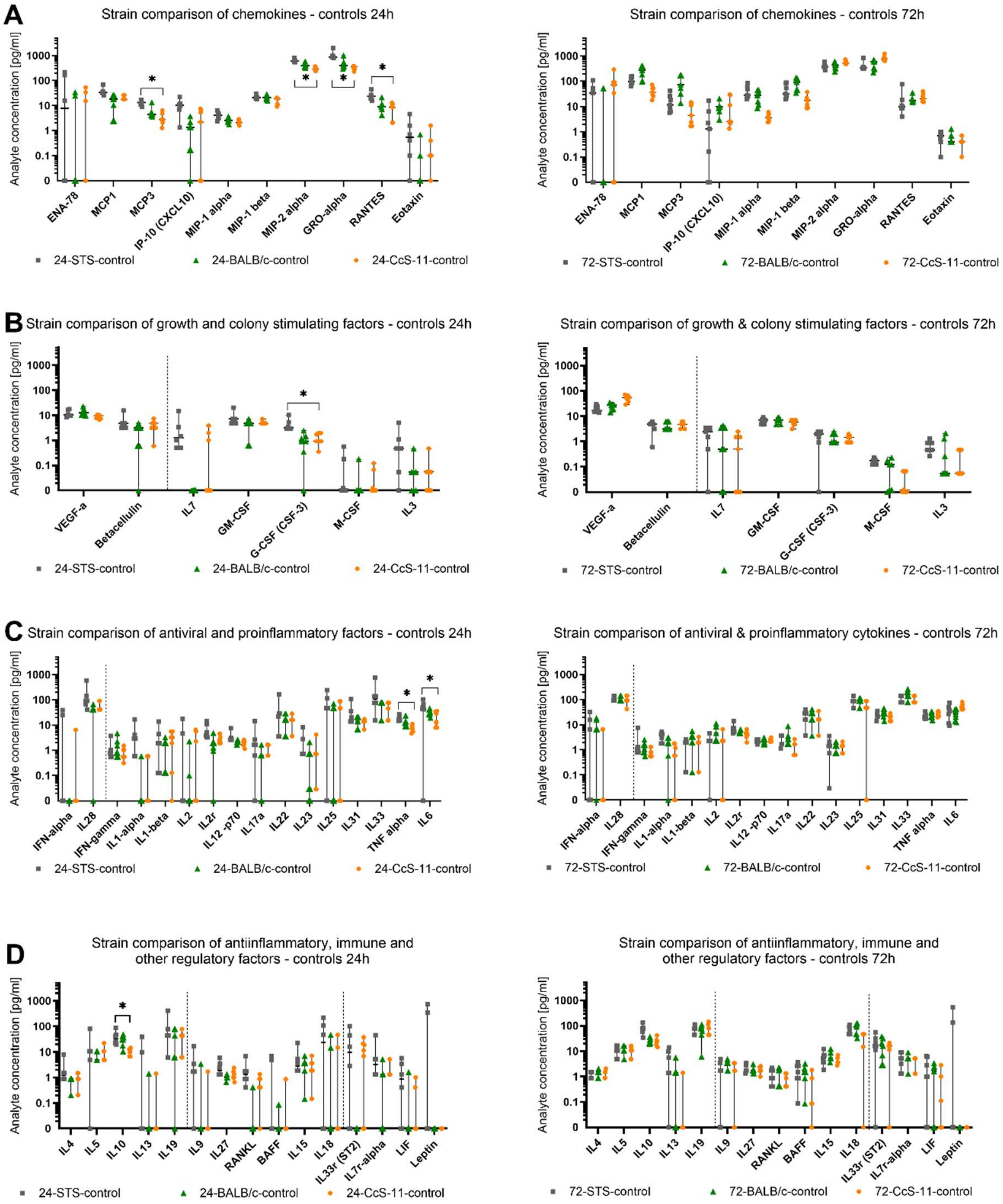

Supplementary Figure 13 | Strain comparison of analyte concentrations (median, 95% CI, logarithmic scale) in supernatants of **uninfected (control) STS, BALB/c, and CcS-11** mouse strain-derived macrophage cultures. Concentrations were determined in five biological replicates for STS and six replicates for CcS-11 and BALB/c derived cultures using Luminex MagPix technology. Statistically significant differences based on the Mann-Whitney multiple comparison test with two-stage step-up multiple comparison corrections (Benjamini, Krieger, Yekutieli; Q=1%) are indicated by asterisks. **(A)** chemokines, **(B)** growth and colony-stimulating factors, **(C)** antiviral and proinflammatory factors, **(D)** antiinflammatory cytokines, immune and other regulatory factors; concentrations reached after 24 hours of cultivation on the left, concentrations reached after 48 h of cultivation (right).

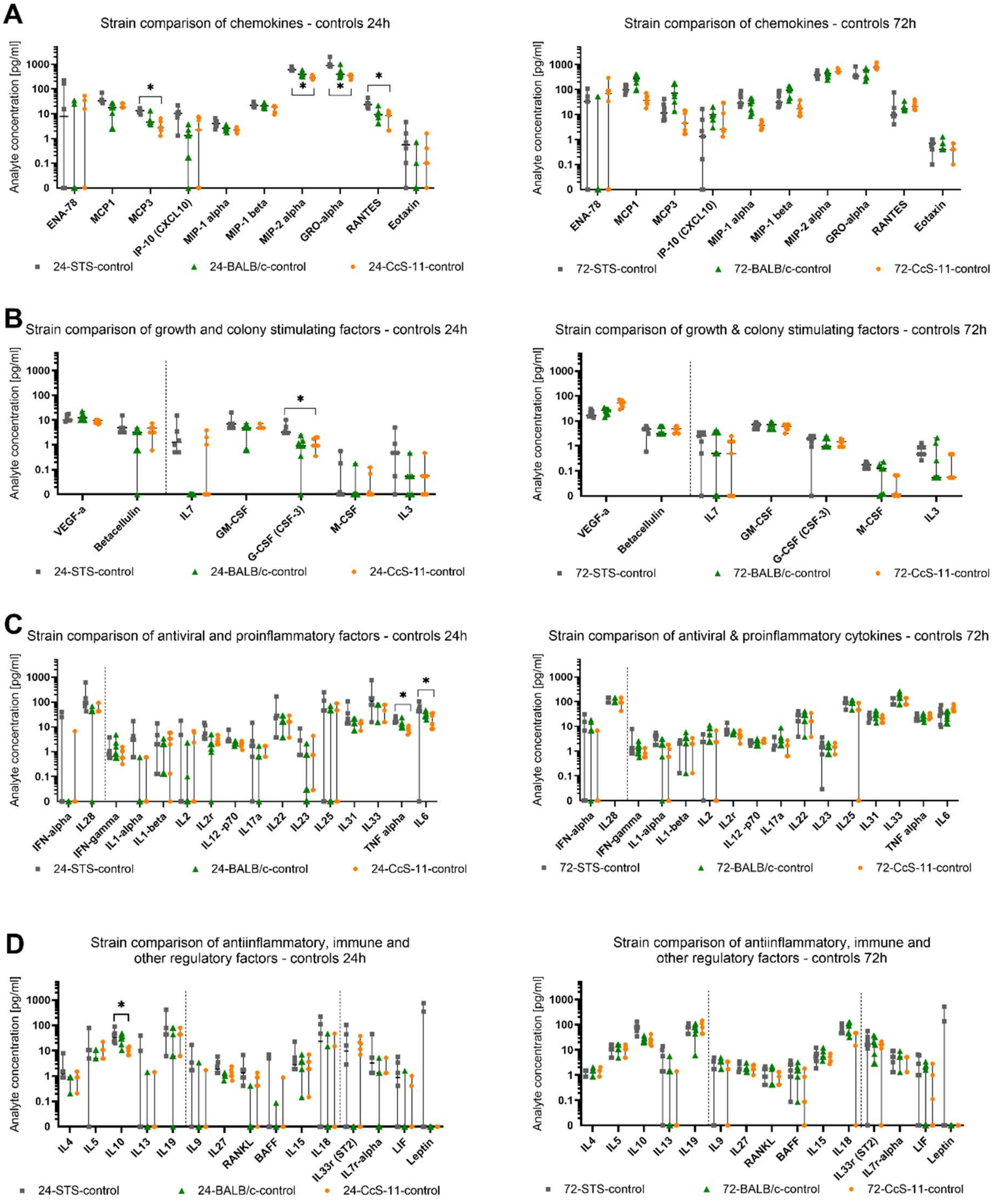

Supplementary Figure 14 | Strain comparison of analyte concentrations (fold change compared to uninfected control, logarithmic scale) in supernatants of **TBEV infected STS, BALB/c, and CcS-11** mouse strain-derived macrophage cultures. Concentrations were determined in five biological replicates for STS and six replicates for CcS-11 and BALB/c derived cultures using Luminex MagPix technology. Statistically significant differences based on the Mann-Whitney multiple comparison test with two-stage step-up multiple comparison corrections (Benjamini, Krieger, Yekutieli; Q=1%) are indicated by asterisks. **(A)** chemokines, **(B)** growth and colony-stimulating factors, **(C)** antiviral and proinflammatory factors, **(D)** antiinflammatory cytokines, immune and other regulatory factors; concentrations reached 24 hours post-infection on the left, concentrations reached 48 h post-infection on the right.

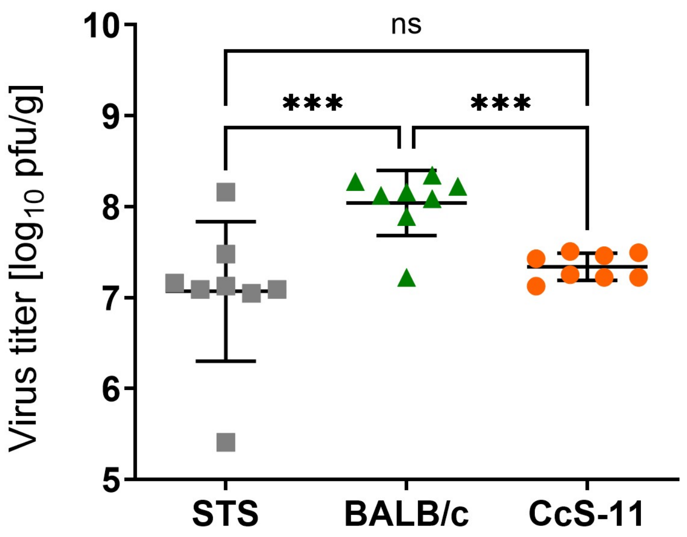

Supplementary Figure 15 | TBEV titers in brains of STS, BALB/c, and CcS-11 mice. Mice were inoculated intracerebrally with 10 pfu of TBEV (strain Neudoerfl). At 6 days post-infection, the brains were collected and subjected to plaque assay. ***, $p < 0.001$.

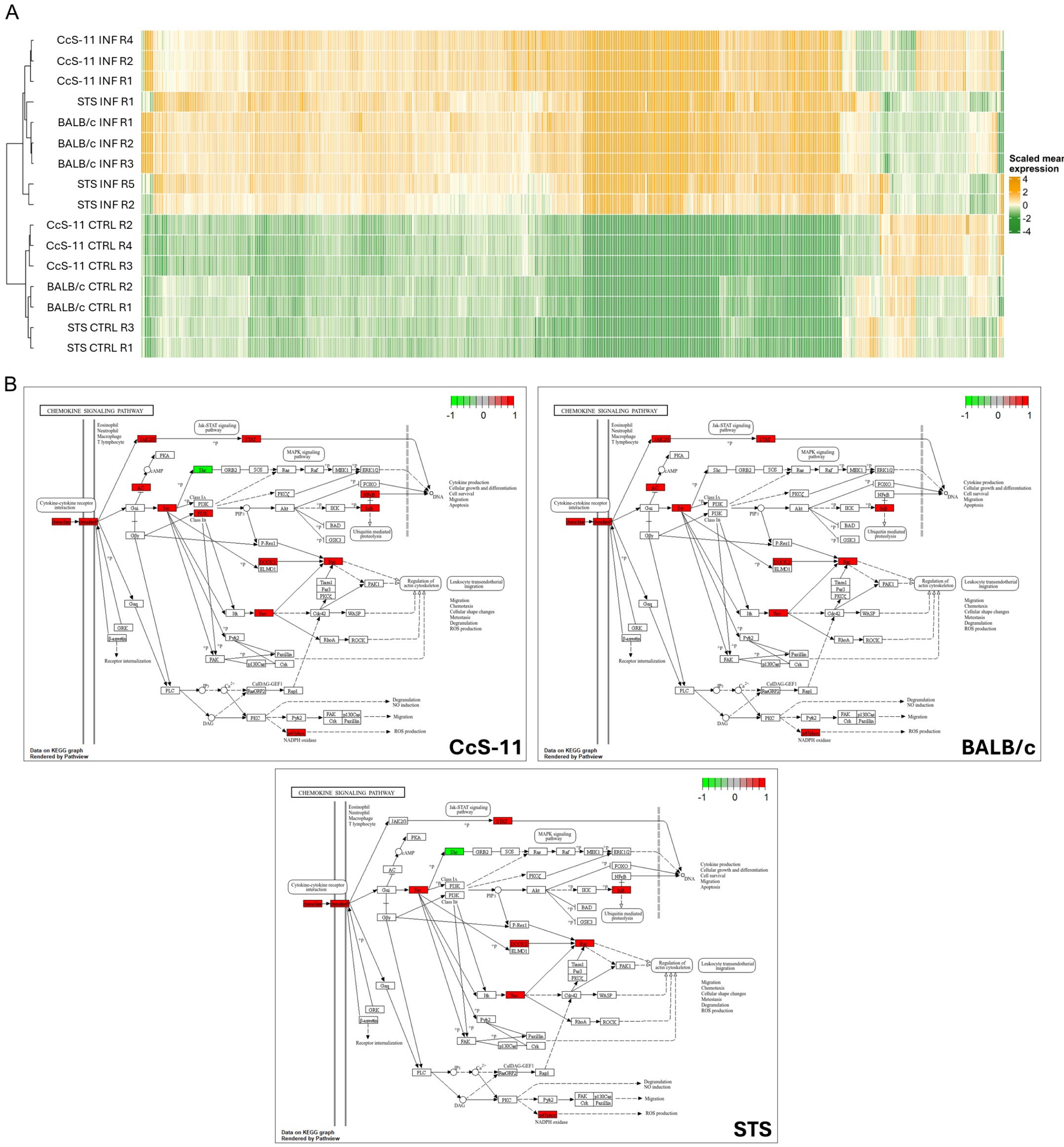

Supplementary Figure 16 | (A) Heatmap of expression of top 500 variable genes across all samples. Samples and genes are clustered based on similar expression patterns. (B) Visualization of chemokine response in KEGG pathways database (mmu04062; [1]) in all mouse strains. Regulation of its components is highlighted in colorscale.

References

1. Kanehisa M, Goto S: **KEGG: kyoto encyclopedia of genes and genomes.** *Nucleic Acids Res* 2000, **28:**27-30.
